# Ionophilic Ru-SNS Complexes as Dual-Function Catalysts for CO_2_ Hydrogenation and Formic Acid Dehydrogenation

**DOI:** 10.1021/acs.inorgchem.5c03184

**Published:** 2025-10-22

**Authors:** Gabriela I. Matiello, Cecília A. Silveira, Gustavo Chacón, Hubert K. Stassen, Jackson D. Scholten

**Affiliations:** † Institute of Chemistry, 28124 UFRGS, Av. Bento Gonçalves, 9500, Agronomia, CEP, Porto Alegre-RS 91501-970, Brazil; ‡ Institute of Chemical Technology (ITQ), Universitat Politècnica de València, Consejo Superior de Investigaciones Científicas (UPV-CSIC), Av. de los Naranjos s/n, Valencia 46022, Spain

## Abstract

This work presents homogeneous Ru complexes containing sulfur-based ionophilic ligands that exhibit high dual catalytic activity for CO_2_ hydrogenation and formic acid dehydrogenation under mild conditions. Indeed, the ionophilic [RuCl_2_(SNS)­(PPh_3_)] complexes promote the reduction of CO_2_ to formate using DBU as a base, either in organic solvents (CH_3_CN or CH_3_CN/THF mixture, achieving TOFs of up to 1500 h^–1^) or in ionic liquids ([BMIm]­[NTf_2_], [OMIm]­[NTf_2_]) at 80 °C and 40 bar (20 bar CO_2_, 20 bar H_2_). Additionally, independent reactions demonstrated that formic acid can be dehydrogenated with TONs of up to 1540 at the same temperature. Notably, one-pot recycling experiments revealed that the ionophilic Ru complexes are also capable of promoting CO_2_ hydrogenation to formate and its subsequent dehydrogenation at 80 °C, providing evidence that these ionophilic Ru complexes are active sulfur-based catalysts for enabling a versatile cycle of hydrogenation/dehydrogenation reactions.

## Graphical abstract

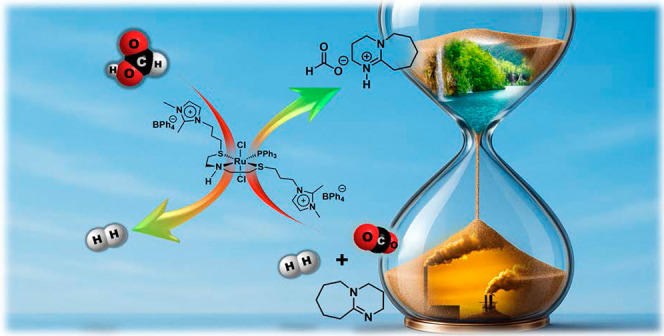

## Introduction

Carbon dioxide is an abundant, low-cost and nontoxic compound that serves as one of the most important raw materials in chemical processes.^1^ Indeed, the transformation of CO_2_ into higher value-added products is of great interest to industry and fundamental for sustainable development. Since CO_2_ is considered a harmful gas with a significant contribution to the greenhouse effect, several systems for capturing CO_2_ have been reported in the literature.^2–4^ In this context, the development of effective catalytic systems capable of converting CO_2_ under mild conditions and allowing for easy manipulation of the catalysts remains challenging. Particularly, the use of transition metal complexes as catalysts for the hydrogenation of CO_2_ to formic acid/formate^5–9^ and methanol^10–12^ has been widely explored. Ruthenium complexes containing phosphines as ligands are commonly used as catalysts in these reactions (Figure 1).^5,6,8,10,11,13–18^ Despite their effectiveness for this purpose, difficult synthetic procedures often involving manipulation under an inert atmosphere are necessary. Alternatively, the use of sulfur analogous ligands (SNS-type) that can coordinate to the metal through sulfur and nitrogen atoms is promising. These SNS ligands have proven to be efficient in the ethylene trimerization reaction^19^ and in the hydrogenation of esters,^20–22^ but their application in the hydrogenation of CO_2_ is still incipient in the literature when compared to phosphines. For example, one study reported that a Ru-SNS complex, even when employed with polyamines and basic additives, was not active for methanol production from CO_2_ hydrogenation, which corroborates the challenging nature of this transformation.^11^

**Figure 1:**
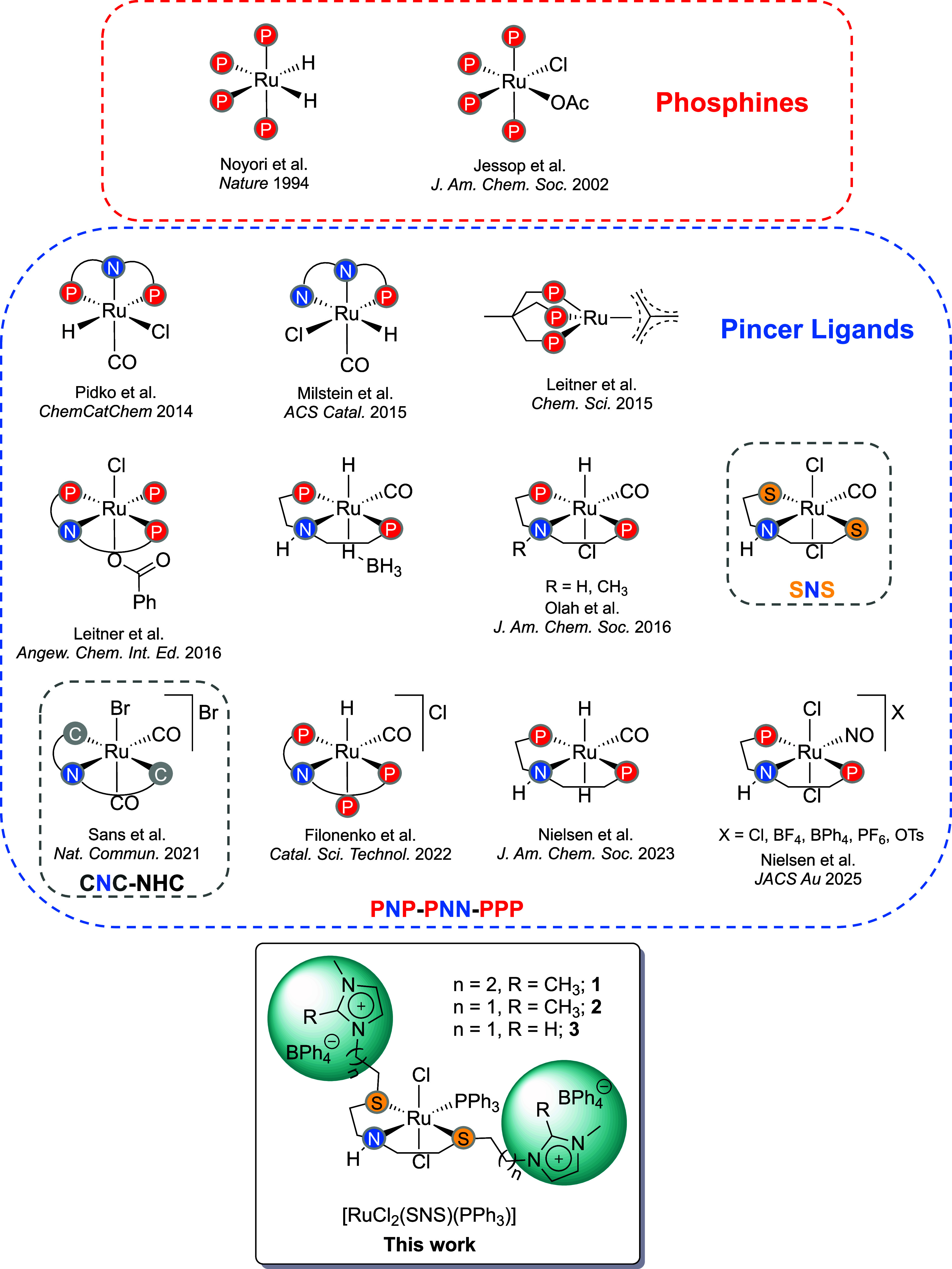
Examples of Ru complexes reported in the literature for CO_2_ hydrogenation (see refs 5,6,8,10,11, and 13–18 for details) and the new ionophilic Ru complexes ([RuCl_2_(SNS)­(PPh_3_)] **1**–**3**) developed in this work.

In recent years, metal complexes containing ionophilic ligands have shown to be a very useful tool in homogeneous catalysis.^23–25^ Ionophilic ligands contain an ionic side chain, which can be used as molecular probes for detecting reaction intermediates through ESI-MS (Electrospray Ionization Mass Spectrometry).^24,25^ Ionophilic metal complexes are widely used as catalysts in liquid–liquid biphasic systems, where one of the phases is an ionic liquid (IL).^23^ In these systems, the ionophilic complex can be retained in the IL phase, allowing for the recycling of the catalytic system and significantly reducing the leaching of the metal into the organic phase. Notably, catalytic systems based on ILs have already been used in the hydrogenation of CO_2_,^26–28^ suggesting that the development of ionophilic metal complexes is a promising approach for creating new and active CO_2_ hydrogenation catalysts. Moreover, it is highly desirable for a catalyst applied in the hydrogenation reaction to also be active in the reverse process, i.e., the dehydrogenation reaction. In fact, among the various compounds used as hydrogen sources, formic acid (FA) has been widely investigated due to its chemically immobilized hydrogen content and low toxicity.^29–36^ Therefore, it is of great interest that the same catalytic system might perform both the hydrogenation of CO_2_ to FA/formate and the dehydrogenation of FA to release H_2_, thus completing a useful hydrogenation/dehydrogenation cycle.

In this work, we describe the use of imidazolium-based SNS ligands (SNS = thioamine) for the preparation of novel ionophilic Ru complexes that exhibit high dual activity in CO_2_ hydrogenation and FA dehydrogenation under mild conditions (Figure 1). Our preliminary results show that the catalytic system based on the Ru-SNS complex **1** produces high yields of formate at 80 °C and 40 bar (20 bar CO_2_/20 bar H_2_), both in organic solvents (achieving TOFs of up to 1500 h^–1^) and in ILs. In addition, this homogeneous complex **1** is capable of catalyzing the dehydrogenation of FA at 80 °C with TONs of up to 1540. Particularly, an initial investigation of the one-pot CO_2_ hydrogenation/formate dehydrogenation catalyzed by the ionophilic Ru-SNS complex **1** provides evidence for the feasibility of a reversible hydrogenation/dehydrogenation cycle.

## Experimental Section

### General

When not mentioned otherwise, all manipulations were performed under air. Infrared spectra were obtained on a Bruker Alpha-P FTIR spectrometer equipped with an ATR (attenuated total reflectance) module. NMR spectra were recorded on a Varian MR400 and a Bruker Ascend400. ^1^H and ^13^C chemical shifts were measured and reported relative to the solvent peaks. For ^31^P NMR analysis, phosphoric acid was used as the standard. Exact masses were determined using high-resolution mass spectrometry with electrospray ionization (ESI-MS) on a Micromass Q-Tof Micro and a Bruker Impact II, using an acetonitrile/methanol solution. Gas chromatography analyses were performed on a MicroGC QMicro DynamiQ-S instrument equipped with a thermal conductivity detector (TCD), a VICI Valco injector, a molecular sieve column for H_2_ and CO analysis (carrier gas: argon, injection temperature: 120 °C, column temperature: 60 °C), and a U-bond column for CO_2_ analysis (carrier gas: helium, injection temperature: 120 °C, column temperature: 70 °C). The 3-(3-bromopropyl)-1,2-dimethyl-1*H*-imidazolium bromide,^37^ 2,2′-dichlorodiethylamine hydrochloride,^38^ [RuCl_2_(PPh_3_)_3_],^39^ [BMIm]­[NTf_2_], and [OMIm]­[NTf_2_][Bibr ref40] have been synthesized according to literature procedures. RuCl_3_·*x*H_2_O was purchased from Sigma-Aldrich. All other compounds and anhydrous-grade solvents were obtained from Alfa Aesar. Acetonitrile was further distilled over P_2_O_5_, degassed by bubbling argon, and stored in a Schlenk flask. Tetrahydrofuran was further distilled in a multisolvent dry-cleaning machine and stored under argon in a Schlenk flask. CO_2_ (4.8) and H_2_ (5.0) gases were purchased from Air Liquide.

### Synthesis of Compounds

#### Isothiouronium Salts

In a typical reaction, the isothiouronium salt was prepared according to a previously reported procedure.^25^ Thiourea (10 mmol) was added to a solution of 3-(3-bromopropyl)-1,2-dimethyl-1*H*-imidazolium bromide (10 mmol) in ethanol (95%, 20 mL). The mixture was refluxed for 16 h, and the solvent was removed in a rotary evaporator until white crystals precipitated. The crystals were then filtered and washed with ethyl ether. Yield: 89%.

^1^H NMR (400 MHz, DMSO-*d*_6_) δ ppm: 2.09 (m, 2H), 2.64 (s, 3H), 3.27 (t, 2H), 3.78 (s, 3H), 4.27 (t, 2H), 7.70 and 7.73 (dd, 2H, *J*_1_ = 13.6 Hz, *J*_2_ = 2 Hz), 9.14 (br s, 4H).

^13^C NMR (100 MHz, D_2_O) δ ppm: 10.23, 27.29, 29.50, 35.33, 46.48, 121.15, 122.95, 145.07, 169.88.

The other isothiouronium salts were prepared using a similar experimental procedure.

### SNS Ligands

#### SNS Ligand (*n* = 2, R = CH_3_)

In a typical reaction, a solution of *S*-3-propyl-2,3-dimethylimidazolium thiouronium dibromide (1.080 g, 2.90 mmol) and KOH (0.407 g; 7.25 mmol) in 10 mL of water was boiled at 100 °C for 30 min. After cooling to room temperature, dichlorodiethylamine hydrochloride (0.259 g, 1.45 mmol) was added to the reaction mixture, and the solution was warmed to 100 °C for an additional 30 min. The solution was then cooled to room temperature, and a solution of NaBPh_4_ (1.240 g, 3.62 mmol) in 10 mL of water was added dropwise, resulting in the immediate precipitation of a solid (Scheme S1). The reaction mixture was stirred at room temperature for 1 h. The solid was filtered and washed three times with 10 mL of water, three times with 10 mL of diethyl ether, and dried under vacuum. The solid is soluble in acetonitrile, acetone, and DMSO. Yield: 1.324 g (1.26 mmol, 87%).

^1^H NMR (400 MHz, DMSO-*d*_6_) δ ppm: 7.59 (d, *J* = 2.1 Hz, 2H), 7.56 (d, *J* = 2.0 Hz, 2H), 7.19 (m), 6.93 (t, *J* = 7.4 Hz), 6.80 (t, *J* = 7.2 Hz), 4.11 (t, *J* = 7.1 Hz, 4H), 3.67 (s, 6H), 2.71 (t, *J* = 6.6 Hz, 4H), 2.59 (t, *J* = 6.7 Hz, 4H), 2.51 (s, 6H), 2.01–1.88 (m, 4H).

^13^C NMR (100 MHz, DMSO-*d*_6_) δ ppm: 164.55, 164.06, 163.57, 163.08, 144.82, 136.00 (d, *J* = 1.1 Hz), 125.78 (d, *J* = 2.7 Hz), 124.03–120.55 (m), 48.74, 46.79, 35.10, 29.57, 27.82, 9.63.

^13^C NMR (APT) (100 MHz, DMSO-*d*_6_) δ ppm: 163.82 (q, J = 49,9 Hz, BPh_4_^–^), 144.86, 136.01 (BPh_4_^–^), 125.82 (BPh_4_^–^), 125.79 (BPh_4_^–^), 125.77 (BPh_4_^–^), 125.74 (BPh_4_^–^), 122.88, 121.99 (BPh_4_^–^), 121.21, 48.87, 46.82, 35.13, 31.72, 29.62, 27.86, 9.67.

#### SNS Ligand (*n* = 1, R = CH_3_)

In a typical reaction, a solution of the isothiouronium salt (1.0 mmol) and KOH (3.0 mmol) in 5 mL of water was boiled at 100 °C for 30 min. After cooling to room temperature, dichlorodiethylamine hydrochloride (0.5 mmol) was added to the reaction mixture, and the solution was warmed to 100 °C for an additional 30 min. The solution was then cooled to room temperature, and a solution of NaBPh_4_ (1.25 mmol) in 6 mL of water was added dropwise, resulting in the immediate precipitation of a solid (Scheme S1). The reaction mixture was stirred at room temperature for 1 h. The solid was filtered and washed three times with 5 mL of water, three times with 5 mL of diethyl ether, and dried under vacuum. Yield: 0.407 g (0.39 mmol, 80%).

^1^H NMR (400 MHz, DMSO-*d*_6_) δ ppm: 7.61 (d, *J* = 2.1 Hz, 2H), 7.55 (d, *J* = 2.1 Hz, 2H), 7.18 (m), 6.93 (t, *J* = 7.4 Hz), 6.80 (t, *J* = 7.2 Hz), 4.26 (t, *J* = 6.7 Hz, 4H), 3.69 (s, 6H), 2.89 (t, *J* = 6.7 Hz, 4H), 2.69 (t, *J* = 6.5 Hz, 4H), 2.62 (t, *J* = 6.1 Hz, 4H), 2.54 (s, 6H).

#### SNS Ligand (*n* = 1, R = H)

In a typical reaction, a solution of the isothiouronium salt (1.0 mmol) and KOH (3.0 mmol) in 5 mL of water was boiled at 100 °C for 30 min. After cooling to room temperature, dichlorodiethylamine hydrochloride (0.5 mmol) was added to the reaction mixture, and the solution was warmed to 100 °C for an additional 30 min. The solution was then cooled to room temperature, and a solution of NaBPh_4_ (1.25 mmol) in 6 mL of water was added dropwise, resulting in the immediate precipitation of a solid (Scheme S1). The reaction mixture was stirred at room temperature for 1 h. The solid was filtered, washed three times with 5 mL of water, three times with 5 mL of diethyl ether, and dried under vacuum. Yield: 0.403 g (0.405 mmol, 81%).

^1^H NMR (400 MHz, DMSO-*d*_6_) δ ppm: 9.01 (s, 2H), 7.70 (d, *J* = 1.7 Hz, 2H), 7.62 (d, *J* = 1.7 Hz, 2H), 7.18 (m), 6.93 (t, *J* = 7.4 Hz), 6.80 (t, *J* = 7.2 Hz), 4.30 (t, *J* = 6.7 Hz, 4H), 3.80 (s, 6H), 2.93 (t, *J* = 6.6 Hz, 4H), 2.69 (t, 4H), 2.60 (t, *J* = 6.3 Hz, 4H).

#### Ionophilic Ru Complexes [RuCl_2_(SNS)­(PPh_3_)]

All manipulations were performed using standard Schlenk techniques. A solution of [RuCl_2_(PPh_3_)_3_] (0.452 g, 0.44 mmol) and the SNS ligand (0.524 g, 0.50 mmol) in 10 mL of acetonitrile was boiled in a sealed tube for 24 h (Scheme S1). After cooling to room temperature, the bright yellow solution was filtered, the solvent was evaporated, and the solid was dried under vacuum. The bright yellow solid was washed six times with diethyl ether and six times with hexane, and then dried under vacuum. The complex is soluble in acetonitrile, acetone, and DMSO.

#### [RuCl_2_(SNS)­(PPh_3_)] **1**

FTIR (cm^–1^): 3135 (NH), 1579, 1477, 1430, 1069, 733, 696, 610 (CS), 529, 513.

^31^P NMR (162 MHz, DMSO-*d*_6_) δ ppm: (a) 45.96, 45.07, 42.28, 28.23, 25.53, −6.88. (b) 46.62, 45.77, 43.06, 31.38, 29.01, 26.29, 24.24, −6.10.

ESI-MS: *m*/*z* calcd C_86_H_92_B_2_ClN_5_PRuS_2_ (M^+^ – Cl), 1448.5455; exp., 1448.5574.

#### [RuCl_2_(SNS)­(PPh_3_)] **2**

FTIR (cm^–1^): 3121 (NH), 1578, 1479, 1427, 1091, 740, 697, 611 (CS), 514.

^31^P NMR (162 MHz, DMSO-*d*_6_) δ ppm: 46.06, 44.87, 43.31, 31.68, 29.31, 27.32, 24.36, −5.91, −7.60.

ESI-MS: *m*/*z* calcd C_86_H_91_B_2_ClN_6_PRuS_2_ (M^+^ – Cl + CH_3_CN), 1461.5402; exp., 1461.5405.

#### [RuCl_2_(SNS)­(PPh_3_)] **3**

FTIR (cm^–1^): 3130 (NH), 1578, 1479, 1427, 1091, 731, 695, 608 (CS), 516.

^31^P NMR (162 MHz, DMSO-*d*_6_) δ ppm: 45.81, 44.67, 43.05, 31.36, 29.00, 26.27, −6.10.

ESI-MS: *m*/*z* calcd C_84_H_85_B_2_ClN_6_PRuS_2_ (M^+^ – Cl + CH_3_CN), 1433.5089; exp., 1433.5114.

### Hydrogenation Reactions

In a typical hydrogenation reaction, DBU (0.240 g, 1.57 mmol), the catalyst (10 μmol), and the chosen solvent (10 mL CH_3_CN, 7 mL CH_3_CN/3 mL H_2_O, 5 mL CH_3_CN/5 mL THF, or 5 mL of ionic liquid) were added to the internal glass of a Parr reactor, which was then sealed. The system was purged three times with CO_2_, after which 20 bar of CO_2_ and 20 bar of H_2_ were introduced into the reactor. The reactor was heated to the desired temperature (50–90 °C), and the reaction mixture was stirred at 150 rpm for the specified time (2–16 h). After this period, the reaction mixture was cooled to room temperature using an ice bath, and an aliquot of the gas phase was analyzed by GC-TCD. The reactor was then depressurized, and the solution was analyzed by ^1^H NMR to determine the yield of formate.

### Dehydrogenation Reactions

In a typical dehydrogenation reaction, formic acid (10 mmol, 0.460 g), the catalyst (5 or 10 μmol) and the IL 1-(diethylaminoethyl)-2,3-dimethylimidazolium *N*-bis­(trifluoromethanesulfonyl)­imide (0.5 or 1 mmol, 0.238 or 0.476 g) were added to the inner glass vessel of a Fischer–Porter reactor. The reactor was then sealed, heated to 80 °C, and the reaction mixture stirred at 400 rpm for 20 h. After this period, the reaction mixture was cooled to room temperature, and an aliquot of the gas phase was analyzed by GC-TCD. The reactor was then depressurized, and a sample of the liquid phase was collected for ^1^H NMR analysis.

### Recycling Experiments

The recycling tests were conducted through one-pot reversible hydrogenation/dehydrogenation reactions. Initially, DBU (0.240 g, 1.57 mmol), [RuCl_2_(SNS)­(PPh_3_)] **1** (10 μmol), and [BMIm]­[NTf_2_] (5 mL) were added to a Parr reactor, which was then sealed and pressurized with 20 bar of CO_2_ and 20 bar of H_2_. The reaction mixture was heated to 80 °C and stirred for 4 h. Afterward, the reactor was cooled to room temperature and depressurized, and the yield of [DBUH]­[HCO_2_] was determined by ^1^H NMR. The solution mixture was then subjected to the dehydrogenation reaction at 80 °C for 19 h. Subsequently, the reactor was cooled to room temperature, depressurized, and an aliquot of the solution was removed and analyzed by ^1^H NMR.

### Quantification by NMR

#### Hydrogenation Reactions

The catalytic reactions were studied and quantified by ^1^H NMR spectroscopy using approximately 700 μL samples taken from the reaction mixtures without dilution. To the samples, 100 μL of DMSO-*d*_6_ was added for the ^2^H lock and 10 μL of DMF was added as an internal standard to calculate the conversion to formate. For reactions in CH_3_CN, water (500 μL) was added to homogenize the reaction mixture before sampling. For reactions in ionic liquids, a sample of the reaction mixture (150–200 mg) was diluted in CD_3_CN (300 μL), and no internal standard was needed, as quantification was performed using the absolute value of the peak integration for the C2–H signal of the imidazolium cation as a standard. ^1^H NMR analyses were performed using 32 scans, a 20 s relaxation delay, and a pulse angle of 90°.

#### Dehydrogenation Reactions

Samples of the reaction mixture (20 mg) were collected both before and after the reaction and diluted in 600 μL of DMSO-*d*_6_. The absolute integration values of the C4–H and C5–H signals of the imidazolium cation were used as internal standards. Conversion was calculated from the integrals of the formate hydrogen signal before and after the reaction. ^1^H NMR analyses were performed using 32 scans with a relaxation delay of 20 s.

### Theoretical Calculations

The study was conducted using Gaussian 16 with the DFT B3LYP/6-311++G­(d,p) level of theory for geometric optimization in all cases.^41^ Single-point calculations were performed at the HF/6-31­(d) level to obtain properties from the optimized geometries.^42^ The molecular electrostatic potential was calculated using the CHELPG algorithm, and the images were generated using the VMD software.^43,44^ The theoretical calculations were carried out considering only the reported compound, without accounting for solvent effects.

## Results and Discussion

First, the imidazolium-based SNS ligands were prepared from the isothiouronium salt using a procedure previously reported by our group.^25^ The ionophilic Ru complexes were synthesized by mixing the SNS ligand and [RuCl_2_(PPh_3_)_3_] in acetonitrile at 80 °C for 24 h (Scheme S1). Notably, after the workup process, the metal complex seems to be stable under air, as its yellow color remains unchanged for several days. The Ru complexes were characterized by FTIR, ^31^P NMR, and electrospray mass spectrometry (ESI-MS). Infrared analysis of complex **1** showed a characteristic C–S stretching band at 610 cm^–1^, which is slightly shifted compared to the 600 cm^–1^ observed in the SNS ligand (Figures S1 and S2). This shift was also observed in complexes **2** and **3** when compared with their respective ligands (Figures S3–S6), indicating the coordination of the sulfur atom to the Ru center. The ^31^P NMR analysis of complex **1** reveals the presence of three isomers (45.96, 45.07, and 42.28 ppm) and triphenylphosphine (−6.88 ppm) in solution (Figure S10a). This complex can exist in three isomeric forms, differing from each other due to the orientation of the S–C_3_Im group relative to the plane of the SNS ligand (*mer*-[RuCl_2_(SNS)­(PPh_3_)]).^20^ Furthermore, the results suggest the existence of complexes in which the SNS ligand is coordinated to the metal center in a bidentate rather than a tridentate fashion, a situation that may occur in the presence of coordinating solvents during sample preparation. Since two chloride ligands are bound to the metal center, two isomeric forms of the complex with a bidentate SNS ligand may exist, originating either from the *mer*- or the *fac*-[RuCl_2_(SNS)­(PPh_3_)] isomer. In one isomer, the chlorides adopt a *trans* configuration, most likely corresponding to the more intense signal at 28.23 ppm. In the other, the chlorides are *cis* to each other, likely giving rise to the weaker signal at 25.53 ppm. In the *cis* configuration, one chloride is positioned *trans* to the PPh_3_ ligand, which may enhance its lability and promote the dissociation of PPh_3_ from the coordination sphere of the metal, thereby reducing the concentration of this isomer. The formation of alternative Ru species in solution lacking coordinated SNS ligands cannot be excluded, given the lability of the SNS ligand in coordinating solvents. Finally, it is not possible to exclude that one of the signals in this region corresponds to the presence of triphenylphosphine oxide in solution. However, depending on the conditions, such as the residual water content in the solvent during synthesis or NMR analysis, the ^31^P NMR spectrum may display additional signals, which could correspond to Ru species with coordinated water molecules (Figure S10b), similar to those observed in a previous study.^45^ The ^31^P NMR spectra for the Ru complexes **2** and **3** are shown in Figures S11 and S12. Similar to what was observed for Ru complex **1**, the ^31^P NMR signals in the 43–46 ppm region for Ru complexes **2** and **3** correspond to three isomers arising from different orientations of the S–C_2_Im group relative to the plane of the SNS ligand. Additionally, the signals observed in the 24–31 ppm region are likely attributed to isomers resulting from the arrangement of chloride ligands around the metal center (*cis*/*trans* configurations), as well as to Ru species containing coordinated water molecules. The presence of triphenylphosphine oxide in the medium also cannot be excluded in these cases. As previously mentioned, these complexes exhibit fluxional behavior in solution due to the labile nature of the SNS ligand. The ESI-MS technique was crucial in confirming the proposed structure of the ionophilic Ru complexes. The ESI­(+)-MS of **1** revealed signals at *m*/*z* = 1448 and *m*/*z* = 1164, corresponding to the cationic compound resulting from the loss of a chloride ligand and another structure resulting from the loss of an anion BPh_4_^–^, respectively (Figure 2). Moreover, the isotopic model corroborates the experimental data, suggesting the presence of the desired ionophilic Ru complex. To obtain more information on the fragmentation of the structures, MS–MS analysis of the peak *m*/*z* = 1164 revealed the peak at *m*/*z* = 902, which may be associated with the loss of phosphine, and a peak at *m*/*z* = 866, resulting from the subsequent loss of HCl (Figure S13). These peaks can also be observed in the mass spectrum shown in Figure 2. For the ESI-MS of Ru complexes **2** and **3**, see Figures S14 and S15. To complement the characterization of the SNS ligands, theoretical calculations were performed to determine the HOMO and LUMO Frontier orbitals. For the SNS ligand containing imidazolium rings in *syn* and *anti*-positions, the HOMO is localized over the sulfur atoms, while the LUMO and LUMO+1 are preferentially located over the imidazolium rings (Figure S16). These results contrast with those observed for the respective nonionophilic SNS ligands, where both HOMO and LUMO are located on the sulfur atoms (Figure S19). For comparison, substituting the methyl group with a hydrogen atom at the C2 position of the imidazolium ring did not alter the locations of the HOMO and LUMO in the SNS ligand (Figure S17). In addition, the effect of the anion on the structure of the SNS ligand was studied using bromide as a model anion. The calculations revealed that the anions stay close to the C2–H at the same H–Br distance, and the HOMO is located on the bromide, not on the sulfur, as observed in the SNS ligand without the anion. Meanwhile, the LUMO remains over the imidazolium ring (Figure S18). This behavior may also be observed for the BPh_4_^–^ anion. Complementary data for the investigated SNS compounds (structural parameters and electrostatic properties) can be seen in Tables S1 and S2.

**Figure 2:**
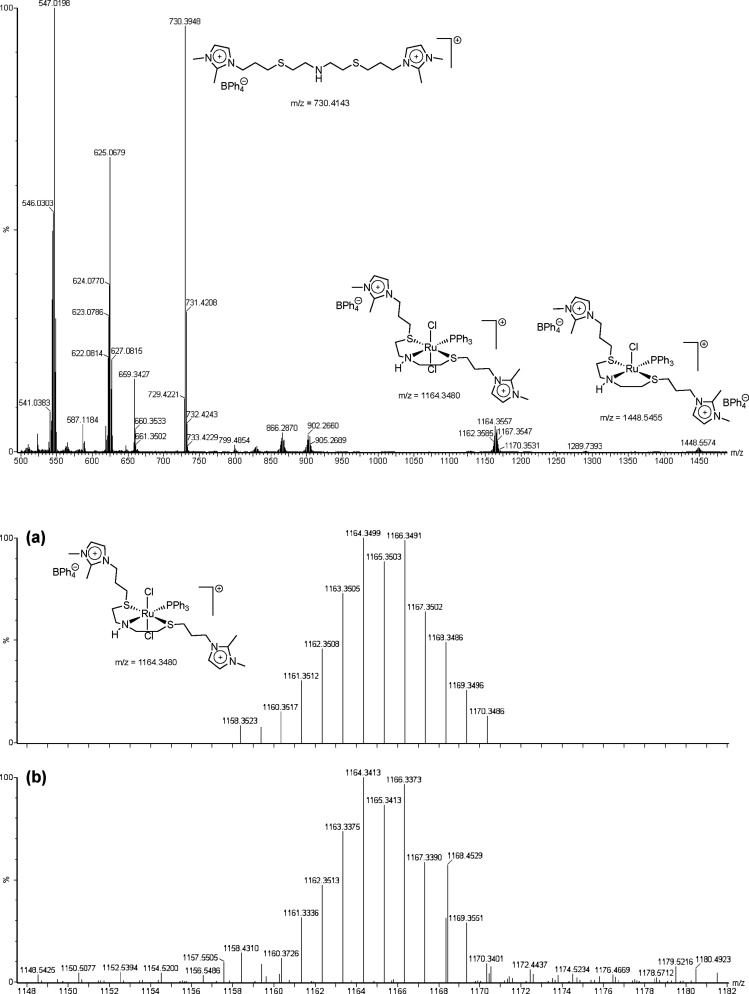
ESI­(+)-MS of the [RuCl_2_(SNS)­(PPh_3_)] complex **1** showing the signals at *m*/*z* = 1448 and 1164 referred to the metal species. (a) Simulated isotopic model and (b) experimental data for the signal *m*/*z* = 1164.

The catalytic activities of the ionophilic Ru complexes [RuCl_2_(SNS)­(PPh_3_)] were evaluated in the hydrogenation of CO_2_. It is noteworthy that all manipulations were conducted in air, which is a significant advantage compared to other conventional air-sensitive catalytic systems. To optimize the reaction conditions, Ru complex **1** was selected as the catalyst precursor. The preliminary reactions were performed using acetonitrile as the solvent to ensure proper solubilization of Ru complex, while DBU (1,8-diazabicyclo[5.4.0]­undec-7-ene) was tested as the base due to its excellent ability to capture CO_2_.^46^ Starting at 50 °C and 40 bar (CO_2_/H_2_ 1:1) in CH_3_CN, the hydrogenation of CO_2_ yielded formate ([DBUH]­[HCO_2_]) in 5% after 5 h (entry 1, Table 1), which is comparable to the use of MeOH as an additive (entry 2, Table 1). Surprisingly, when the reaction was conducted without MeOH but for a longer period (16 h), the product yield increased to 30% (entry 3, Table 1). This suggests that, contrary to previous studies,^6^ the use of an additive is not beneficial for this catalytic system. This effect is also observed with Et_3_N as an additive, as the coordinating nature of both MeOH and Et_3_N is unfavorable to the reaction under these conditions (entries 13 and 15, Table 1). Increasing the temperature to 80 °C, [DBUH]­[HCO_2_] was formed with a 20% yield in CH_3_CN after 5 h (entry 4, Table 1), similar to the yield observed at 50 °C after 16 h (entry 3, Table 1). Maintaining the temperature at 80 °C for 16 h, the yield of [DBUH]­[HCO_2_] increased to 74% (entry 5, Table 1). For the reaction in CH_3_CN, a solid precipitate was observed (Figure S20). This solid was analyzed by ^1^H/^13^C NMR, revealing signals at 4.29 ppm (^1^H, [DBU**H**]^+^), 164.28 ppm (^13^C, N**C**N from [DBUH]^+^), and 159.31 ppm (^13^C, H**C**O_3_^–^), indicating the formation of bicarbonate (Figures S21 and S22). In this case, it is proposed that carbamate is formed during the reaction, and the presence of water subsequently leads to the formation of stable bicarbonate. This is consistent with previously reported studies on the reaction of CO_2_ with bicyclic amidines.^47,48^ A similar equilibrium between carbamate/bicarbonate was observed when acetonitrile containing traces of water was used in the presence of DBU/CO_2_.^49^ These salts are intermediates in the formation of formate. To dissolve the bicarbonate, two solvent mixtures were tested: CH_3_CN/H_2_O and CH_3_CN/THF. However, the reaction in the CH_3_CN/H_2_O mixture was not efficient for obtaining formate (entry 6, Table 1). Analysis of the ^13^C NMR spectra revealed a characteristic peak for the acetate ion at around 179 ppm. This indicates that, in the presence of water, acetonitrile undergoes hydration to form acetamide, which is subsequently hydrolyzed to acetic acid, forming DBU acetate in this case. Therefore, the result indicates that the catalyst is water-tolerant and remains active in both nitrile hydration and amide hydrolysis reactions.^50^ Rather than being highlighted as primary transformations, these side reactions reveal that the Ru-SNS framework preserves its catalytic activity under aqueous conditions, where many metal complexes are typically deactivated. These findings suggest that the ionophilic [RuCl_2_(SNS)­(PPh_3_)] complexes not only facilitate hydrogen transfer but also retain their catalytic performance across different reaction pathways, highlighting their multifunctional character. Indeed, the ability to operate effectively under diverse conditions makes these complexes valuable tools for advancing green chemistry initiatives and promising candidates for broader catalytic applications where water stability is crucial. Alternatively, a CH_3_CN/THF solvent mixture was used, producing formate in up to 51% yield (entries 7–9, Table 1). To our delight, when the reaction was performed in CH_3_CN/THF at 80 °C, excellent yields of [DBUH]­[HCO_2_] (80–98%) were obtained (entries 10, 12, and 14, Table 1). Figure 3a illustrates the performance of the catalytic system over time at 80 °C, showing that after 5 h the product yield reached 91%. GC-TCD analysis of the gas phase revealed the presence of CO_2_ and H_2_, indicating that CO is not formed in this catalytic system (Figure S25). Notably, increasing the pressure to 60 bar (CO_2_/H_2_ 1:1) led to the formation of up to 100% of the formate product after 2 h of reaction (entry 16, Table 1). In particular, changing the pressure ratio of CO_2_/H_2_ to 1:2 reduced the amount of formate to 70% (entry 17, Table 1). This observation is contrary to reports in the literature^51^ and suggests that a lower CO_2_/H_2_ ratio favors the hydrogenation reaction. This discrepancy may be due to a reduced amount of CO_2_ dissolved in the solution with a CO_2_/H_2_ ratio of 1:2, which decreases the availability of carbamate/bicarbonate and, consequently, the conversion to formate. Although reactions with a higher H_2_ ratio are prone to methanol formation, ^1^H NMR analysis of the reaction using a CO_2_/H_2_ ratio of 1:2 confirmed that no methanol was produced under these conditions. Moreover, comparing the hydrogenation of CO_2_ in CH_3_CN/THF at temperatures ranging from 60 to 90 °C (entries 8–11, Table 1), a gradual increase in product yield was observed as expected. Figure 3b shows the amount of formate produced as a function of temperature and the estimation of the apparent activation energy (*E*_a_). The apparent *E*_a_ for the hydrogenation of CO_2_ catalyzed by Ru complex **1** was determined to be 75.93 kJ/mol. To evaluate the nature of the active species in this catalytic system (homogeneous vs heterogeneous), an experiment was performed with the addition of Hg before starting the reaction (entry 18, Table 1). The yield of [DBUH]­[HCO_2_] considerably dropped when compared to the reaction without Hg (entries 14 and 18, Table 1). To assess the possible formation of Ru(0) particles during the reaction, a hydrogenation experiment was performed for 30 min, after which the reaction was stopped and cooled to room temperature. A black solid was observed under these conditions, suggesting that part of the Ru complex had been reduced to metallic Ru. The reaction mixture was then centrifuged to remove the solid, and the experiment was continued for a total of 5 h (entry 19, Table 1). In this case, the yield of [DBUH]­[HCO_2_] was 72%, lower than that obtained under uninterrupted reaction conditions (entries 12 and 19, Table 1). This reduced but still satisfactory [DBUH]­[HCO_2_] yield suggests that, although Ru(0) particles are likely formed in situ and may also act as additional hydrogenation catalysts, the reaction proceeds predominantly via a homogeneous pathway. Notably, the CO_2_ hydrogenation reaction catalyzed by the ionophilic Ru complex **1**, using a large excess of DBU, afforded an initial TOF of 1500 h^–1^ (entry 20, Table 1). In this case, the low formate yield (20%) after 5 h of reaction is likely due to the formation of large amounts of carbamate and bicarbonate, which even precipitate in the reaction medium. The presence of these salts was confirmed by the signals at 145.01 ppm (N**C**O_2_) and 167.86 ppm (N**C**N) from carbamate, while the signals at 160.93 ppm (H**C**O_3_^–^) and 164.94 ppm (N**C**N) correspond to bicarbonate in the ^13^C NMR spectrum (Figure S26). High catalytic activities were also observed for the ionophilic complexes **2** and **3** (entries 21 and 22, Table 1), demonstrating that these Ru complexes are effective catalysts in the hydrogenation of CO_2_. For comparison, the hydrogenation of CO_2_ catalyzed by [RuCl_2_(PPh_3_)_3_] produced a 43% yield of formate at 80 °C after 16 h in CH_3_CN/THF, which represents lower activity than that of the ionophilic [RuCl_2_(SNS)­(PPh_3_)] catalysts (entries 12, 21, and 22, Table 1).

**Table 1:**
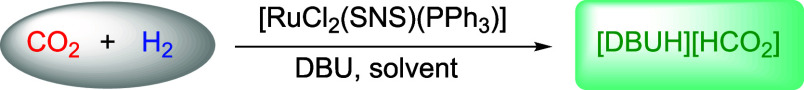
Hydrogenation of CO_2_ Catalyzed by Ionophilic [RuCl_2_(SNS)­(PPh_3_)] Complexes^a^

entry	catalyst	*T* (°C)	*t* (h)	solvent	additive	yield (%)^b^	TON	TOF (h^–1^)
1	**1**	50	5	CH_3_CN		5	8.0	1.6
2	**1**	50	5	CH_3_CN	MeOH	4	6.4	1.3
3	**1**	50	16	CH_3_CN		30	48	3.0
4	**1**	80	5	CH_3_CN		20	32	6.4
5	**1**	80	16	CH_3_CN		74	118	7.4
6	**1**	50	5	CH_3_CN/H_2_O		0.4	0.6	0.1
7	**1**	50	16	CH_3_CN/THF		20	32	2.0
8^c^	**1**	60	2	CH_3_CN/THF		8	8.6	4.3
9^c^	**1**	70	2	CH_3_CN/THF		51	55	27
10^c^	**1**	80	2	CH_3_CN/THF		80	86	43
11^c^	**1**	90	2	CH_3_CN/THF		90	96	48
12	**1**	80	5	CH_3_CN/THF		91	146	29
13	**1**	80	5	CH_3_CN/THF	MeOH	72	115	23
14	**1**	80	16	CH_3_CN/THF		98	157	9.8
15	**1**	80	16	CH_3_CN/THF	Et_3_N	1	1.6	0.1
16^d^	**1**	80	2	CH_3_CN/THF		100	160	80
17^e^	**1**	80	2	CH_3_CN/THF		70	112	56
18	**1**	80	16	CH_3_CN/THF	Hg(0)^f^	47	75	4.7
19^g^	**1**	80	5	CH_3_CN/THF		72	115	23
20^h^	**1**	80	5	CH_3_CN/THF		20 (10)	1000 (500)	200 (1500)
21	**2**	80	5	CH_3_CN/THF		89	142	28
22	**3**	80	5	CH_3_CN/THF		85	136	27

a Reaction conditions: DBU (1.6 mmol), [Ru] (10 μmol), DBU/catalyst = 160, 40 bar (20 bar CO_2_, 20 bar H_2_), solvent (10 mL, mixtures: CH_3_CN/H_2_O 7 mL/3 mL, CH_3_CN/THF 5 mL/5 mL).

b Yield of [DBUH]­[HCO_2_] compared to the base determined by^1^H NMR.

c DBU (1.6 mmol), [Ru] (15 μmol), DBU/catalyst = 107.

d 60 bar (CO_2_/H_2_ 1:1).

e 60 bar (CO_2_/H_2_ 1:2).

f Added at the beginning of the reaction.

g After 30 min, the mixture was centrifuged, and the reaction was then continued to complete a total of 5 h.

h Reaction using DBU (20 mmol), [Ru] (4 μmol), DBU/catalyst = 5000. In parentheses, the values obtained after 20 min of reaction.

**Figure 3:**
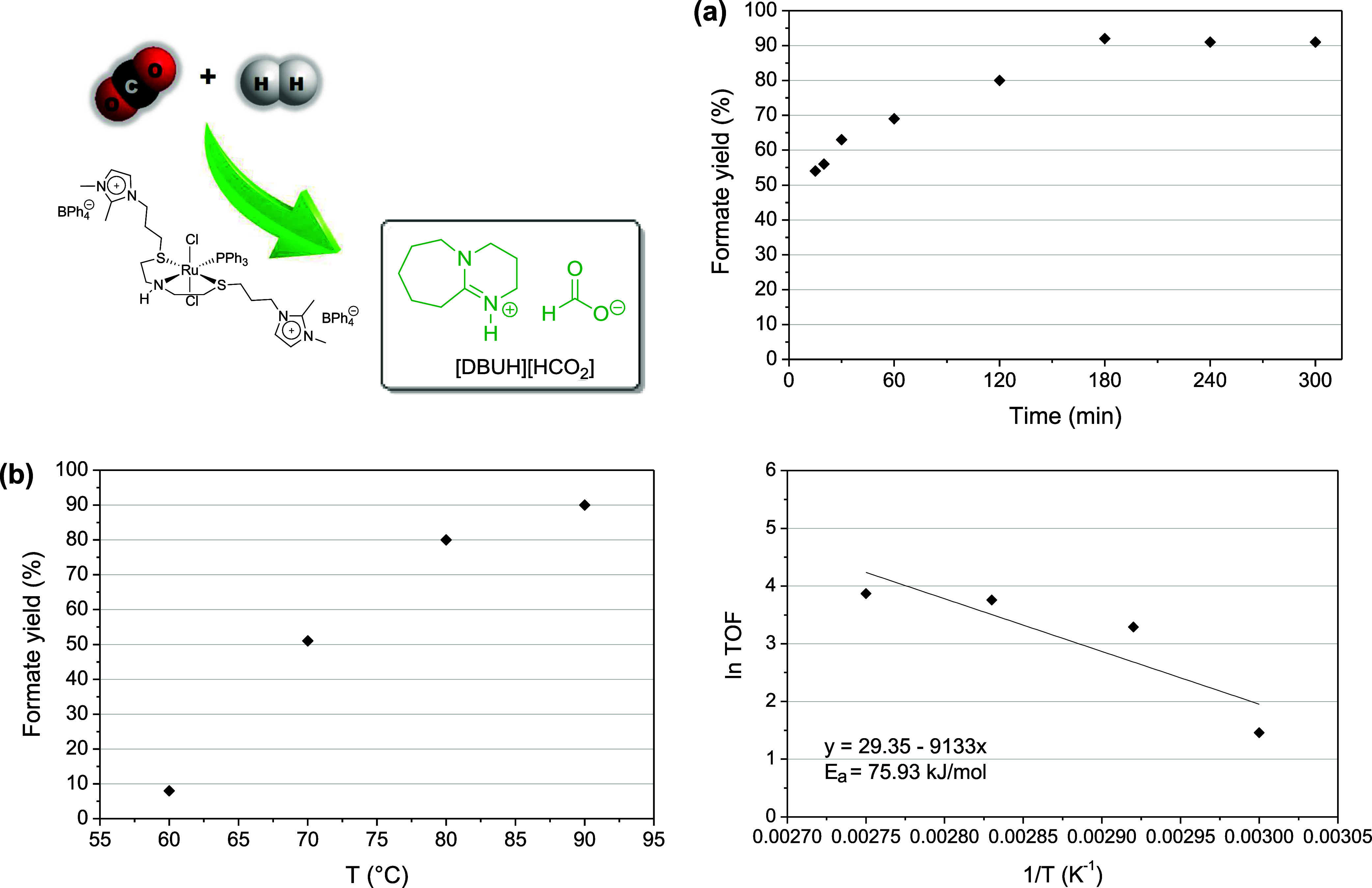
Hydrogenation of CO_2_ catalyzed by Ru complex **1** in CH_3_CN/THF. (a) Formate vs time at 80 °C. (b) Formate vs temperature (reactions for 2 h) and determination of the apparent *E*_a_. Reaction conditions: DBU (1.6 mmol), [Ru] (15 μmol), DBU/catalyst = 107, 40 bar (20 bar CO_2_, 20 bar H_2_).

Generally, metal complexes with sulfur-based ligands provide less catalytic activity for CO_2_ hydrogenation than those containing analogous phosphorus-based ligands.^11^ To explain the remarkable activity of the [RuCl_2_(SNS)­(PPh_3_)] complexes, it was deduced that the ionic moiety of the ligand plays an important role in the catalyst’s performance. It is well-known that ionic liquids (ILs) based on the imidazolium cation are capable of capturing and storing CO_2_ molecules due to their favorable ionic-pair spatial disposition.^52–54^ Applying this ability to the imidazolium-derived SNS ligand may represent the reason for the observed catalytic activity and its absence in other sulfur-containing metal catalysts.^11^ These arguments provide strong evidence for the importance of the ionic group in assisting the CO_2_ transformation in the metal catalyst. However, further investigation is desirable to confirm the influence of such SNS ligands in the hydrogenation of CO_2_ catalyzed by the ionophilic Ru complexes. In fact, understanding the specific interactions between the ionic liquid environment and the Ru complexes will be crucial for optimizing the catalytic process and enhancing CO_2_ conversion efficiencies. This optimization could lead to the development of more effective catalysts that not only improve reaction rates but also promote selectivity toward desired products, ultimately contributing to more sustainable chemical processes.

To expand and transform the present catalytic system into a more sustainable process, the hydrogenation of CO_2_ was tested in ILs, which are alternative solvents for environmentally friendly reactions.^55–57^ As advantages, the use of ILs favors the dissolution of the gases (mainly CO_2_) and the immobilization of the ionophilic catalyst. The ILs [BMIm]­[NTf_2_] ([BMIm] = 1-*n*-butyl-3-methylimidazolium) and [OMIm]­[NTf_2_] ([OMIm] = 1-*n*-octyl-3-methylimidazolium) were tested under the optimized conditions (80 °C, 40 bar CO_2_/H_2_ 1:1, DBU/cat = 160, Table 2). The [OMIm]­[NTf_2_] IL was the most efficient reaction medium, primarily due to the longer nonpolar alkyl chain of the imidazolium cation, which enhances the absorption of CO_2_ and H_2_ (entry 3, Table 2). Moreover, GC-TCD analysis of the gas phase from CO_2_ hydrogenation catalyzed by the ionophilic [RuCl_2_(SNS)­(PPh_3_)] **1** complex in [BMIm]­[NTf_2_] indicated that no CO was formed (Figure S27), similar to the reaction performed in CH_3_CN/THF. Notably, a slightly higher amount of formate than one equivalent relative to the base was observed in the reaction conducted in IL (entry 3, Table 2). A higher amount of formic acid relative to the base was reported in a study where mixing formic acid and diisopropylethylamine in a 1:1 ratio generated an ionic phase containing formic acid/formate and diisopropylethylammonium ions in a 2:1 acid/base ratio.^58^ A similar type of interaction, though to a lesser extent, may also be occurring in our catalytic system. In particular, CO_2_ hydrogenation using a biphasic system consisting of an imidazolium-based IL and water led to the formation of an imidazolium formate adduct, which can be solubilized into the aqueous phase depending on the structure of the imidazolium cation.^26^ It is possible that such an imidazolium formate adduct is also present in our case, suggesting that the IL environment may stabilize imidazolium formate species, thereby enhancing their participation in subsequent reaction steps and potentially leading to higher yields of valuable products. Another study demonstrated that CO_2_ hydrogenation in the presence of imidazolium acetate IL can produce formic acid concentrations exceeding the IL concentration, which was explained by stabilization of the acid species through an IL buffering effect.^27^ Therefore, further investigation into the specific interactions between ILs and formic acid/formate species could provide deeper insights into their stabilization mechanisms, ultimately enabling the design of tailored ionic liquid systems to maximize efficiency in CO_2_ hydrogenation reactions.

**Table 2:**
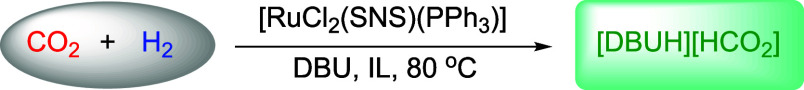
Hydrogenation of CO_2_ Catalyzed by the Ionophilic [RuCl_2_(SNS)­(PPh_3_)] Complexes in ILs^a^

entry	catalyst	IL	*t* (h)	yield (%)^b^	formate/base	formate/IL	TON	TOF (h^–1^)
1	**1**	[BMIm][NTf_2_]	5	87	0.87	0.08	139	28
2	**1**	[BMIm][NTf_2_]	2	67	0.67	0.07	107	54
3	**1**	[OMIm][NTf_2_]	2	100	1.08	0.13	173	86
4	**2**	[BMIm][NTf_2_]	5	66	0.66	0.06	106	21
5	**3**	[BMIm][NTf_2_]	5	60	0.60	0.05	96	19

a Reaction conditions: DBU (1.6 mmol), [Ru] (10 μmol), DBU/catalyst = 160, IL (5 mL), 40 bar (20 bar CO_2_, 20 bar H_2_), 80 °C.

b Determined by ^1^H NMR.

A proposed mechanism for the CO_2_ hydrogenation reaction catalyzed by the ionophilic complex [RuCl_2_(SNS)­(PPh_3_)] (**I**) is outlined in Scheme 1. The process is envisioned to begin with the dissociation of the phosphine ligand from complex **I**, enabling coordination and activation of H_2_ to generate the presumed intermediate **II**. This is followed by the transient dissociation of a sulfur donor atom from the flexible SNS ligand, which may open a coordination site on the ruthenium center for binding to the carbamate and/or bicarbonate, leading to intermediates **IIIa** and **IIIb**, respectively. A hydride transfer step, followed by the subsequent release of DBU (from intermediate **IIIa**) and/or DBU/water (from intermediate **IIIb**), would then yield intermediate **IV**. The catalytic cycle may proceed through a second heterolytic cleavage of H_2_ assisted by the DBU base (intermediates **IV** and **V**), which facilitates the release of formate and the regeneration of intermediate **II**. Alternatively, it is plausible that DBU deprotonates the N–H moiety of the SNS ligand, generating an amido species (intermediate **VI**) that promotes H_2_ activation and formate dissociation, thereby regenerating intermediate **II** through a parallel pathway. In this scenario, the N–H site within the SNS ligand could have a more direct influence on catalytic performance, similar to what is observed in the hydrogenation of CO_2_ catalyzed by Ru-PNP complexes.^11^ While direct experimental evidence for these intermediates is currently lacking, this mechanistic hypothesis is supported by several indirect observations. Notably, the superior catalytic performance of [RuCl_2_(SNS)­(PPh_3_)] compared to Ru complexes with nonionophilic sulfur ligands suggests a beneficial role of the SNS framework. Additionally, the known affinity of CO_2_ for imidazolium-based ILs further supports the idea that the ionic nature of the SNS ligand enhances CO_2_ capture and activation, thereby contributing to the observed reactivity.

**Scheme 1:**
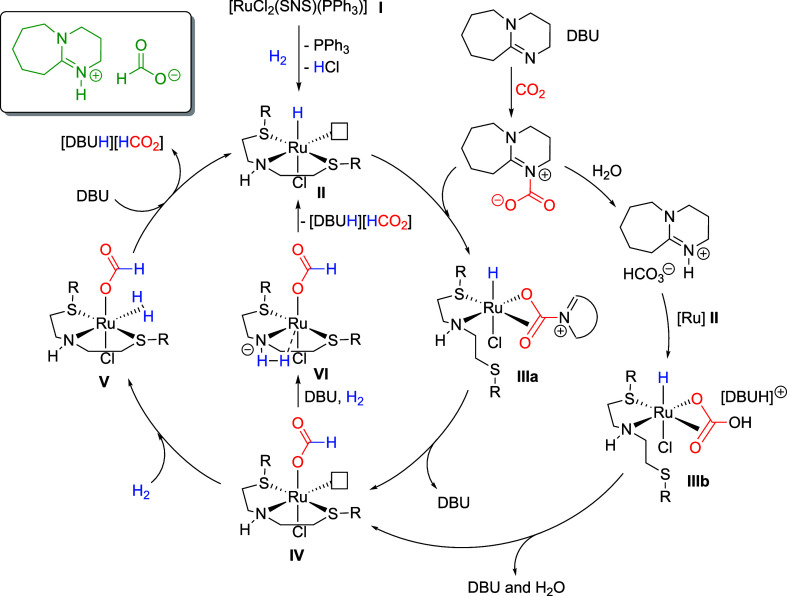
Proposed Mechanism for the Hydrogenation of CO_2_ Catalyzed by the Ionophilic [RuCl_2_(SNS)­(PPh_3_)] Complexes

In light of their promising performance in CO_2_ hydrogenation and the growing interest in systems capable of reversible hydrogen storage, [RuCl_2_(SNS)­(PPh_3_)] complexes **1**–**3** were also examined as catalysts for the dehydrogenation of formic acid (FA). This exploration underscores the versatility of these complexes in catalyzing both hydrogenation and dehydrogenation processes, which is desirable for the development of sustainable energy solutions. This reaction was carried out at 80 °C in the presence of an amine-functionalized IL (1-(diethylaminoethyl)-2,3-dimethylimidazolium *N*-bis­(trifluoromethanesulfonyl)­imide). For the Ru complex **1**, the results showed conversions of up to 77% and TONs ranging from 340 to 1540 (Table 3). Gas-phase analysis by GC-TCD confirmed the presence of H_2_ and CO_2_, indicating FA dehydrogenation (Figure S28). It was observed that the highest conversion was achieved in the presence of AgBF_4_, which was added to induce the formation of a Ru complex with a vacant site (entry 4, Table 3). However, even without this additional agent, the most effective catalytic system was identified as being composed of only 5 μmol of catalyst and 0.5 mmol of the amine-functionalized IL (entry 1, Table 3). Moreover, under the optimized conditions (5 μmol of catalyst, 0.5 mmol IL), Ru complexes **2** and **3** produced 56% conversion (entry 6, Table 3) and 44% conversion (entry 7, Table 3), respectively. These results demonstrate that the same catalyst efficiently promotes both the hydrogenation of CO_2_ and the release of hydrogen gas from FA. This performance is remarkable for Ru complexes with sulfur-based ligands and represents a significant advancement in the field of homogeneous catalysis.

**Table 3:**
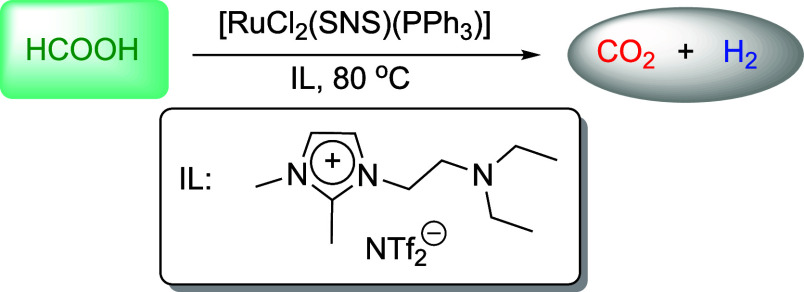
Dehydrogenation of FA Catalyzed by the Ionophilic [RuCl_2_(SNS)­(PPh_3_)] Complexes and an Amine-Functionalized IL^a^

entry	catalyst	*n*_cat_ (μmol)	*n*_IL_ (mmol)	conversion (%)	TON	TOF (h^–1^)
1	**1**	5	0.5	68	1360	68
2	**1**	10	0.5	34	340	17
3	**1**	5	1	44	880	44
4^b^	**1**	5	0.5	77	1540	77
5	**1**	10	1	69	690	34
6	**2**	5	0.5	56	1120	56
7	**3**	5	0.5	44	880	44

a Reaction conditions: FA (10 mmol), 80 °C, 20 h.

b Reaction using AgBF_4_ (10 μmol).

Finally, based on the results showing that the ionophilic Ru-SNS complexes are capable of both CO_2_ hydrogenation and FA dehydrogenation, the one-pot reversible CO_2_ hydrogenation to formate and its subsequent dehydrogenation were performed in the presence of [RuCl_2_(SNS)­(PPh_3_)] **1** at 80 °C in [BMIm]­[NTf_2_] (Figure 4). The reversible cycle of CO_2_ hydrogenation/formate dehydrogenation was carried out for five cycles, with satisfactory product yields achieved at least in the initial two cycles. The successive decrease in catalytic activity may be explained by the continuous reduction of the ionophilic Ru-SNS complex into metallic particles during the recycling reactions. This argument is supported by the observed continuous darkening of the solution, indicating that, over time, the ionophilic Ru-SNS complex acts as a source of likely less active Ru(0) species in the solution, similar to what was observed in the reaction carried out in CH_3_CN/THF (entry 18, Table 1). Such behavior is not surprising, since it is well-known that imidazolium ILs are suitable stabilizing media for the generation of metal particles,^54,57,59,60^ and that the uncoordinated imidazolium-based SNS ligand may also act as an additional stabilizer for the metal particles. Moreover, the drop in the catalytic activity of the system may also be associated with the handling of the experiments conducted in air, which includes the removal of samples for product quantification. Overall, these preliminary recycling results demonstrate the versatile behavior of ionophilic Ru-SNS complexes as active catalysts for reversible hydrogenation and dehydrogenation reactions under mild conditions.

**Figure 4:**
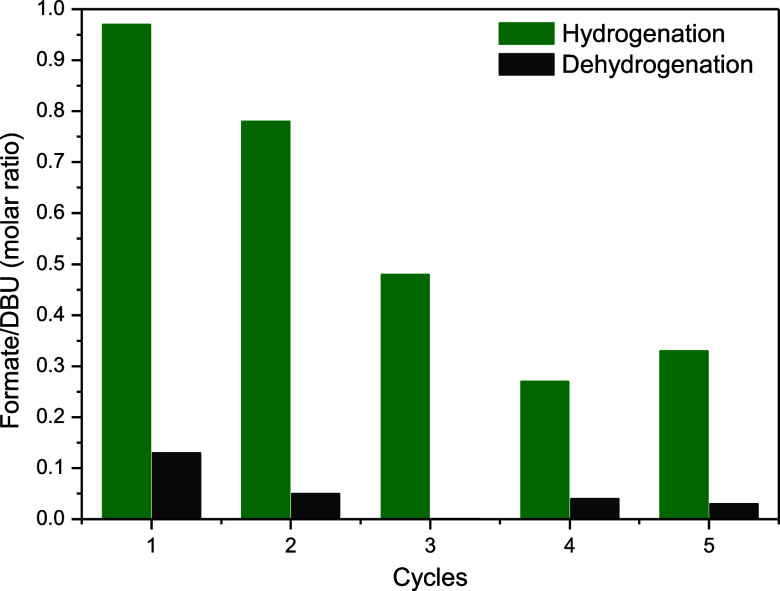
Recycling experiments involving the one-pot reversible CO_2_ hydrogenation and subsequent formate dehydrogenation catalyzed by the ionophilic [RuCl_2_(SNS)­(PPh_3_)] complex **1** in [BMIm]­[NTf_2_]. Reaction conditions: DBU (1.6 mmol), [Ru] (10 μmol), DBU/catalyst = 160, IL (5 mL), 40 bar (20 bar CO_2_, 20 bar H_2_), 80 °C. The yield was determined by ^1^H NMR.

In Table S3, the catalytic activity of the present ionophilic Ru-SNS complexes is compared with other state-of-the-art catalysts for CO_2_ hydrogenation. Our system achieves high conversion of CO_2_ to formate under relatively mild conditions (40–60 bar, CO_2_/H_2_ 1:1, 80 °C, 2–5 h) (entry 1, Table S3). A pioneering Ru-phosphine system exhibited satisfactory activity but required elevated pressure conditions (205 bar, supercritical CO_2_) (entry 2, Table S3).^5^ However, a similar Ru-phosphine catalyst produced a formic acid-to-amine molar ratio of 1.68 under milder conditions (40 bar, 50 °C) in the presence of an alcohol additive (entry 3, Table S3).^6^ In the same context, Ru-PNP systems afforded notable amounts of formic acid/formate but required elevated pressures (up to 120 bar) or temperatures (up to 120 °C) (entries 4 and 7, Table S3).^8,15^ Catalytic approaches targeting methanol synthesis generally required energy-demanding conditions, involving high pressures (up to 80 bar), elevated temperatures (up to 155 °C), and extended reaction times (up to 36 h) (entries 5, 6, 8, and 9, Table S3).^10,11,14^ Notably, the Ru-SNS complex exhibited no activity under such conditions (entry 10, Table S3).^11^ A Ru catalyst bearing a ligand based on *N*-heterocyclic carbene is also effective for CO_2_ hydrogenation to formic acid, but harsh conditions (60 bar and up to 140 °C) together with long reaction times (72 h) are required (entry 11, Table S3).^16^ Although a Ru-PNP system was reported to produce formate under relatively mild conditions (entry 12, Table S3),^17^ more recent Ru-PNP-based systems for formic acid production have shown significant progress, achieving yields of 56–95% at lower temperatures (25–40 °C) (entries 13 and 14, Table S3).^13,18^ In this context, the ionophilic Ru-SNS complexes exhibit satisfactory activity in CO_2_ hydrogenation compared with state-of-the-art catalysts reported in the literature. This performance can be attributed to the incorporation of an imidazolium-based SNS ligand into the Ru complex, which imparts specific catalytic advantages. The ionophilic SNS framework likely creates a CO_2_-affine microenvironment at the metal center and enhances catalyst retention, particularly in ionic liquids, thereby minimizing leaching and enabling recyclability. As a result, the ionophilic Ru-SNS complexes not only exhibit high activity for CO_2_ hydrogenation to formate and for formic acid dehydrogenation, but also promote one-pot reversible CO_2_ hydrogenation/formate dehydrogenation under mild conditions. These features highlight that the role of the ionophilic SNS ligand extends beyond simple coordination, forming part of a multifunctional ligand architecture that enables efficient catalyst operation in both organic solvents and ionic liquid media.

## Conclusions

In summary, this study presents the synthesis of ionophilic Ru complexes containing sulfur-based ligands that exhibit efficient dual catalytic activity in CO_2_ hydrogenation and FA dehydrogenation. The ionophilic [RuCl_2_(SNS)­(PPh_3_)] complexes catalyze the selective transformation of CO_2_ to formate in organic solvents or ILs under mild conditions (80 °C and 40 bar, 20 bar CO_2_/20 bar H_2_). Their activity in CO_2_ hydrogenation may be attributed to the ionic pair of the SNS ligand, which likely generates a CO_2_-affine microenvironment at the metal center, consistent with the known affinity between CO_2_ and imidazolium-based ILs. Moreover, in reactions conducted in ILs, the ionic nature of the SNS ligand promotes favorable confinement of the catalyst within the IL phase, a highly desirable feature in homogeneous systems. The ionophilic Ru-SNS complexes also exhibited satisfactory activity for FA dehydrogenation at 80 °C. Notably, these complexes catalyze one-pot reversible CO_2_ hydrogenation/formate dehydrogenation, thus enabling a versatile hydrogenation/dehydrogenation cycle. These results demonstrate that the new ionophilic [RuCl_2_(SNS)­(PPh_3_)] complexes are simple and highly active dual catalysts for CO_2_ hydrogenation and FA (or formate) dehydrogenation under mild conditions, serving as excellent alternatives to conventional phosphine-based catalysts. Therefore, this work opens new possibilities for the design of novel sulfur-based compounds with high potential as ligands in catalytically active metal complexes.

## Supplementary Material



## Notes

FTIR, ^1^H/^13^C/^31^P NMR, and ESI-MS spectra; theoretical calculations; and GC-TCD analyses (PDF)
