# Supplementary material for: Ionophilic Ru-SNS Complexes as Dual-Function Catalysts for CO2 Hydrogenation and Formic Acid Dehydrogenation
Source: Inorg Chem. 2025 Oct 22;64(43):21429–41. doi: 10.1021/acs.inorgchem.5c03184 (PMC12587404; doi:10.1021/acs.inorgchem.5c03184)
Supplement: Supplementary file 1 [file ic5c03184_si_001.pdf]

**Supporting Information**

# Ionophilic Ru-SNS Complexes as Dual-Function Catalysts for $CO_2$ Hydrogenation and Formic Acid Dehydrogenation

Gabriela I. Matiello,[‡] Cecília A. Silveira,[‡] Gustavo Chacón,[‡#] Hubert K. Stassen,[‡] and Jackson D. Scholten*[‡]

[‡] Institute of Chemistry, UFRGS, Av. Bento Gonçalves, 9500, Agronomia, CEP 91501-970, Porto Alegre-RS.

Corresponding author: jackson.scholten@ufrgs.br

[#] Institute of Chemical Technology (ITQ), Universitat Politècnica de València, Consejo Superior de Investigaciones Científicas (UPV-CSIC), Av. de los Naranjos s/n, 46022, Valencia, Spain.

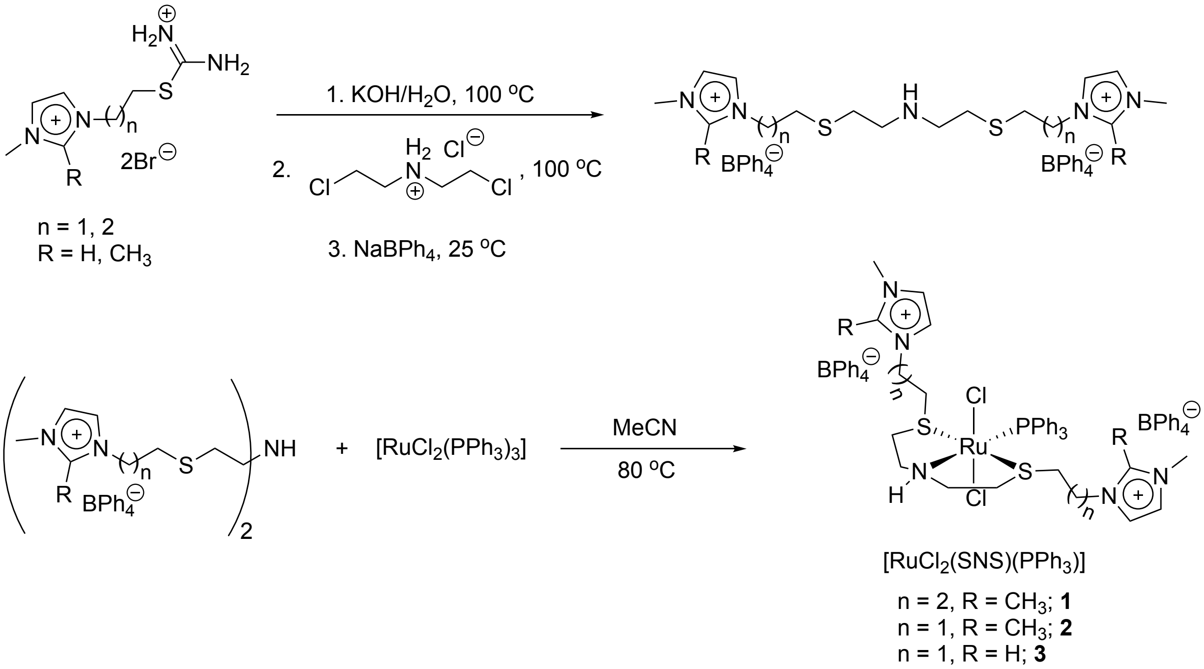


**Scheme S1.** Synthesis of the SNS ligands and the ionophilic Ru complexes $[RuCl_2(SNS)(PPh_3)]$ **1**-**3**.

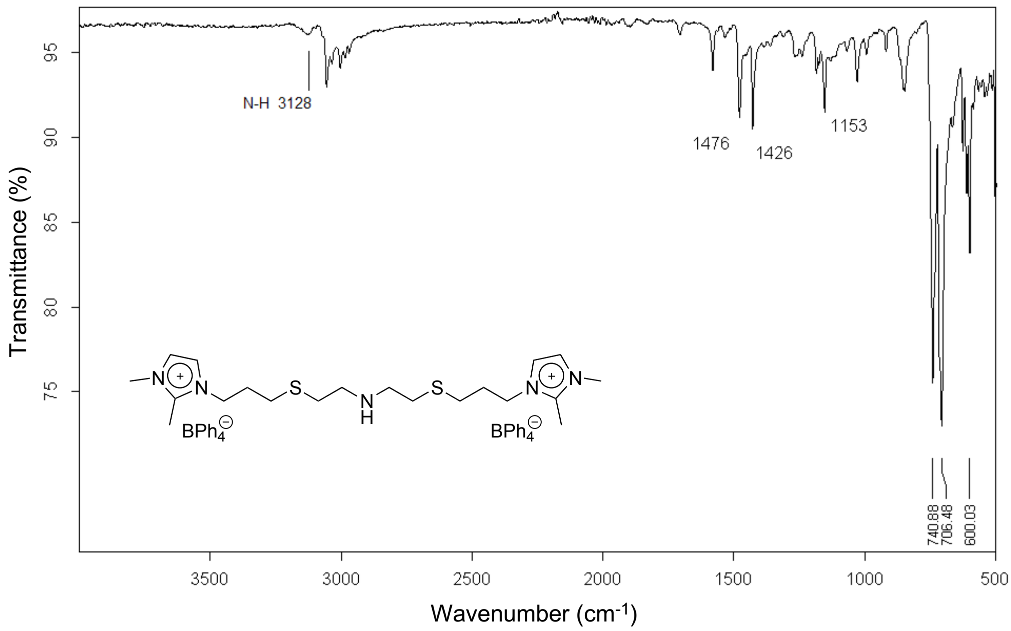


**Figure S1.** FTIR analysis of the SNS ligand (n = 2, R = $CH_3$).

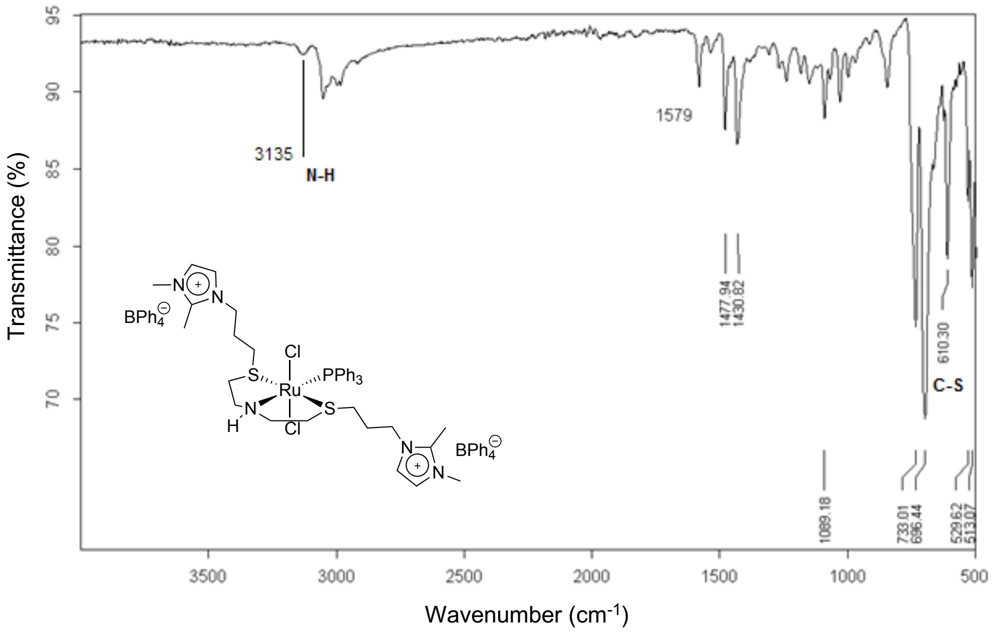


**Figure S2.** FTIR analysis of the ionophilic [$RuCl_2$(SNS)($PPh_3$)] complex **1**.

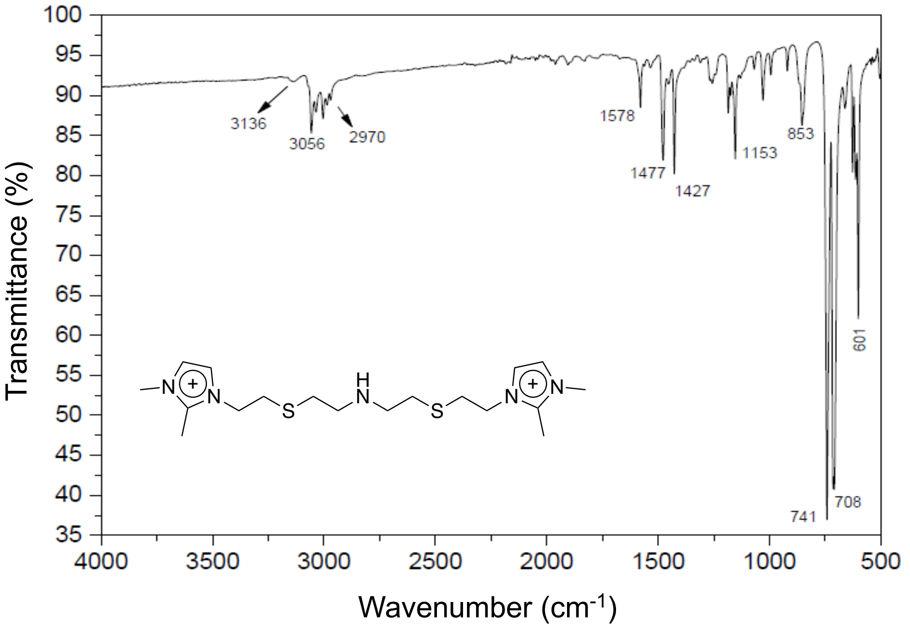


**Figure S3.** FTIR analysis of the SNS ligand (n = 1, R = $CH_3$).

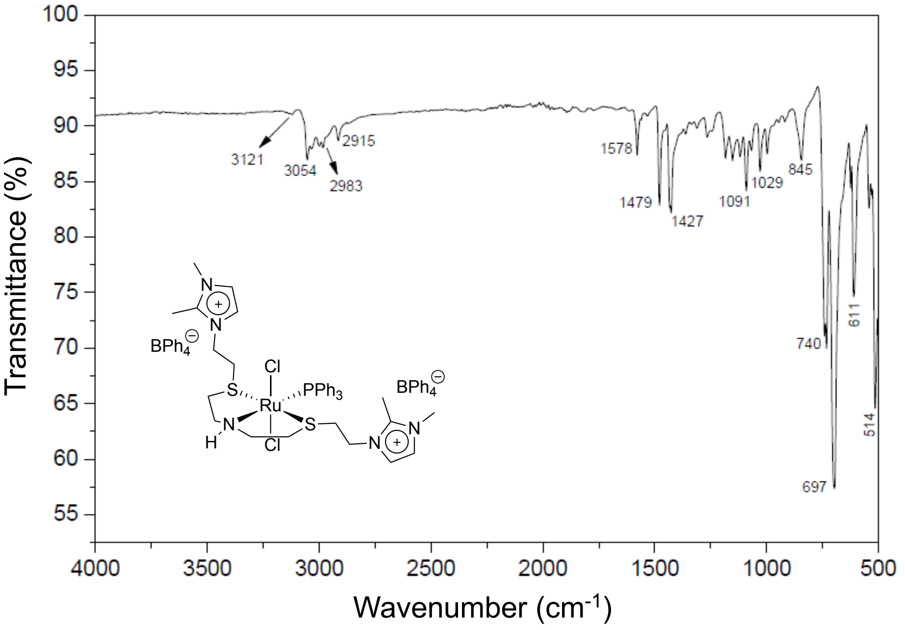


**Figure S4.** FTIR analysis of the ionophilic [$RuCl_2$(SNS)($PPh_3$)] complex **2**.

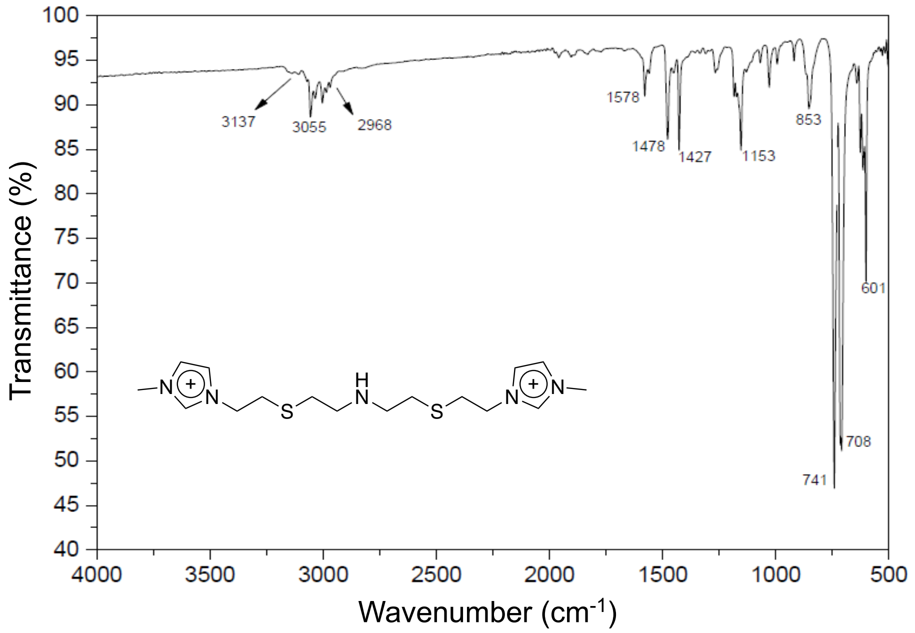


**Figure S5.** FTIR analysis of the SNS ligand (n = 1, R = H).

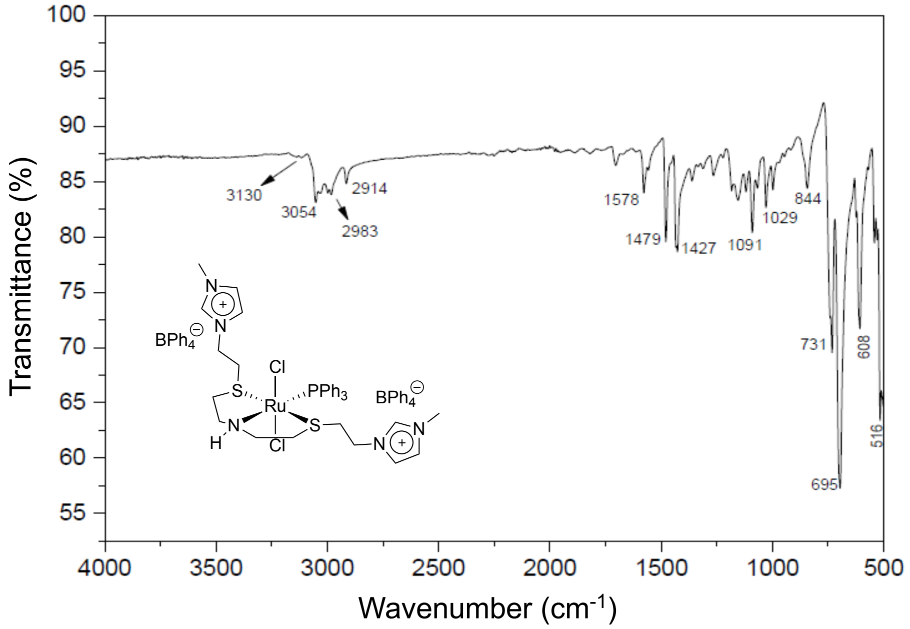


**Figure S6.** FTIR analysis of the ionophilic [$RuCl_2(SNS)(PPh_3)$] complex **3**.

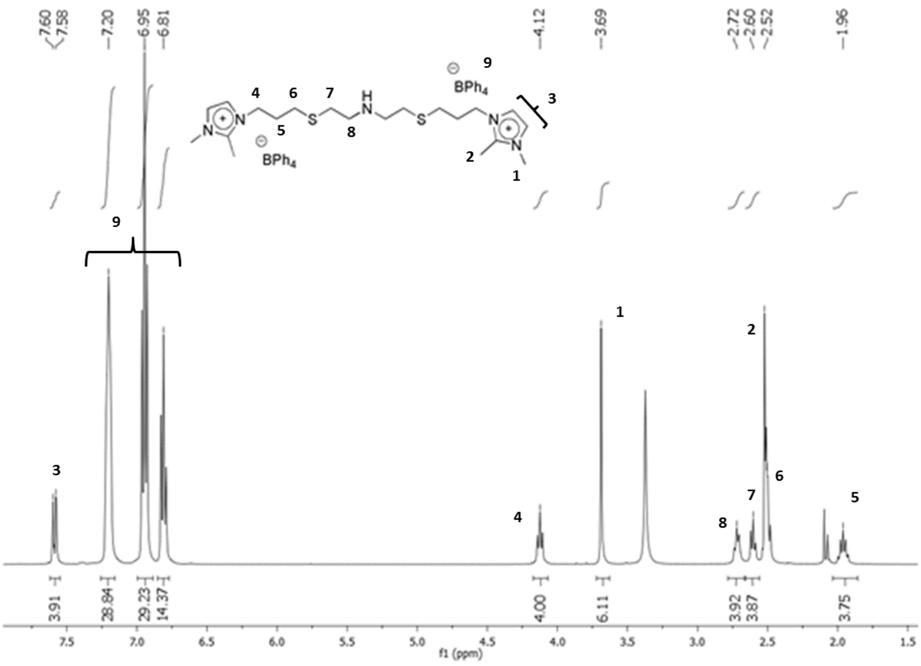


**Figure S7.** $^1$H NMR analysis of the SNS ligand (n = 2, R = $CH_3$) in DMSO-$d_6$.

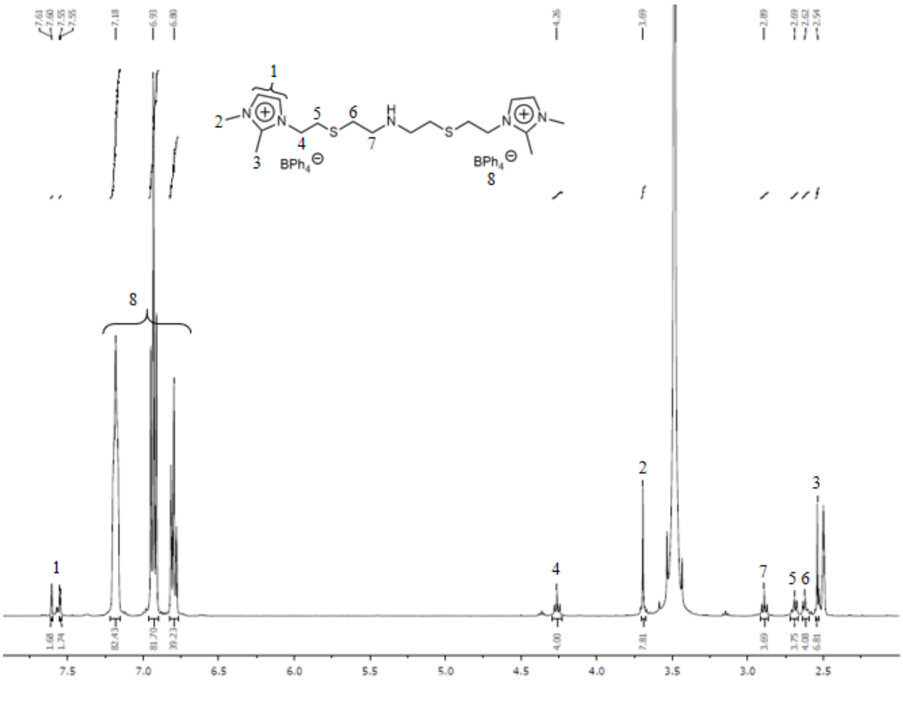


**Figure S8.** $^1$H NMR analysis of the SNS ligand (n = 1, R = $CH_3$) in DMSO-$d_6$.

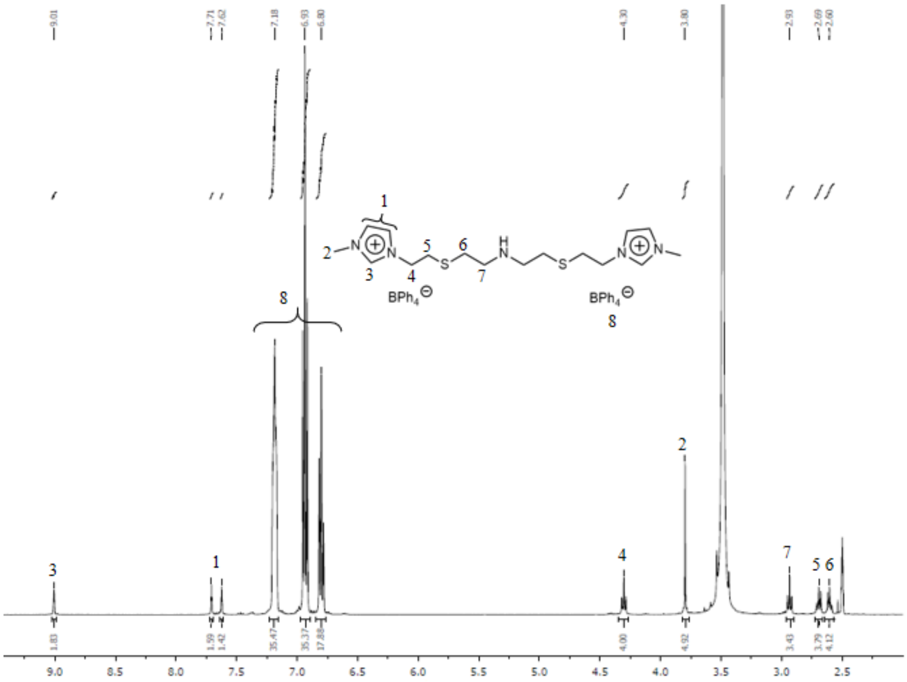

**Figure S9.** $^1$H NMR analysis of the SNS ligand (n = 1, R = H) in DMSO-$d_6$.

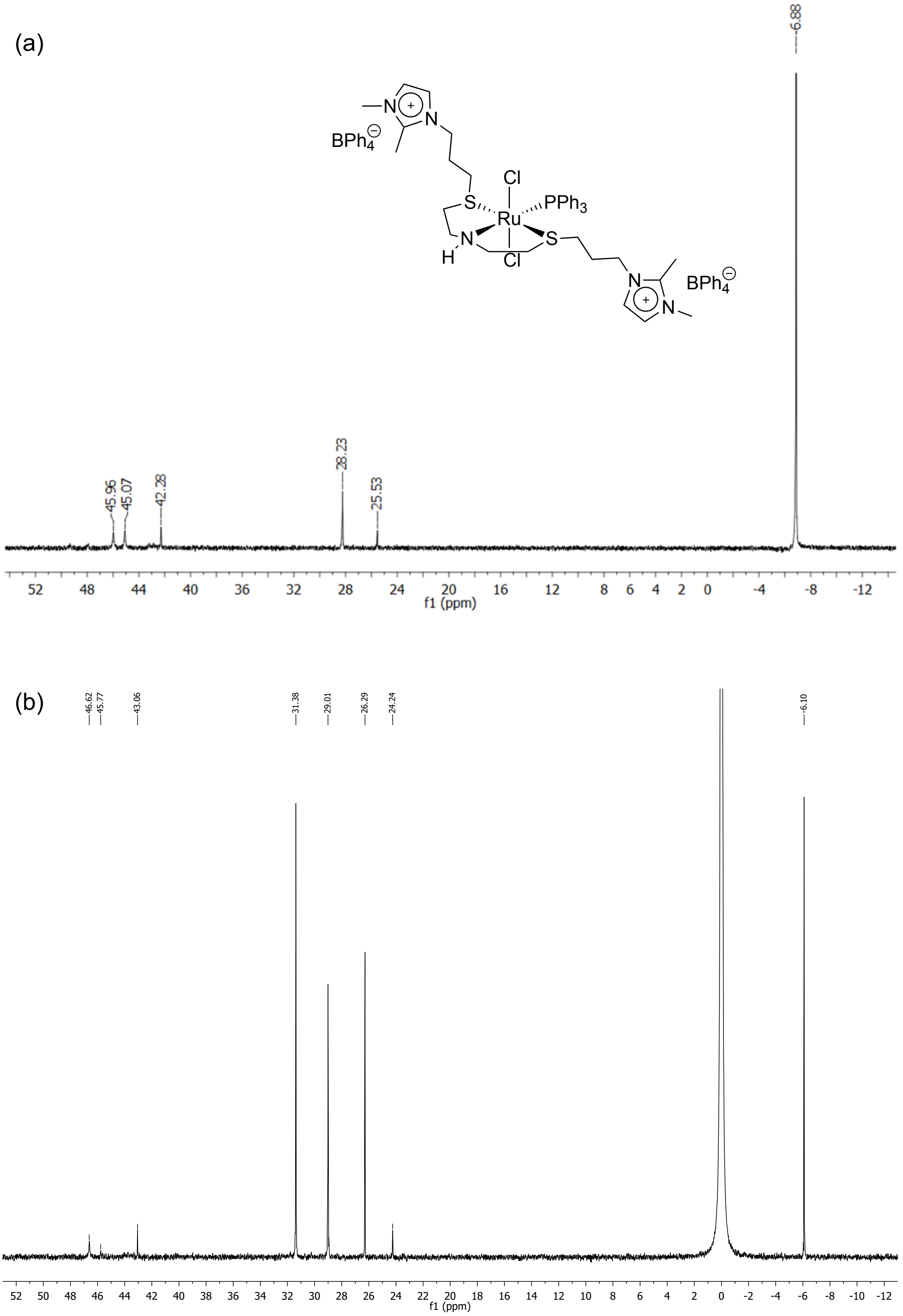


**Figure S10.** $^{31}$P NMR analysis of the ionophilic [$RuCl_2$(SNS)($PPh_3$)] complex **1** in DMSO-$d_6$. (a) Initial synthesis of the Ru complex, and (b) repeated reaction following the same procedure to obtain the Ru complex (in this case, $H_3PO_4$ was used as the standard).

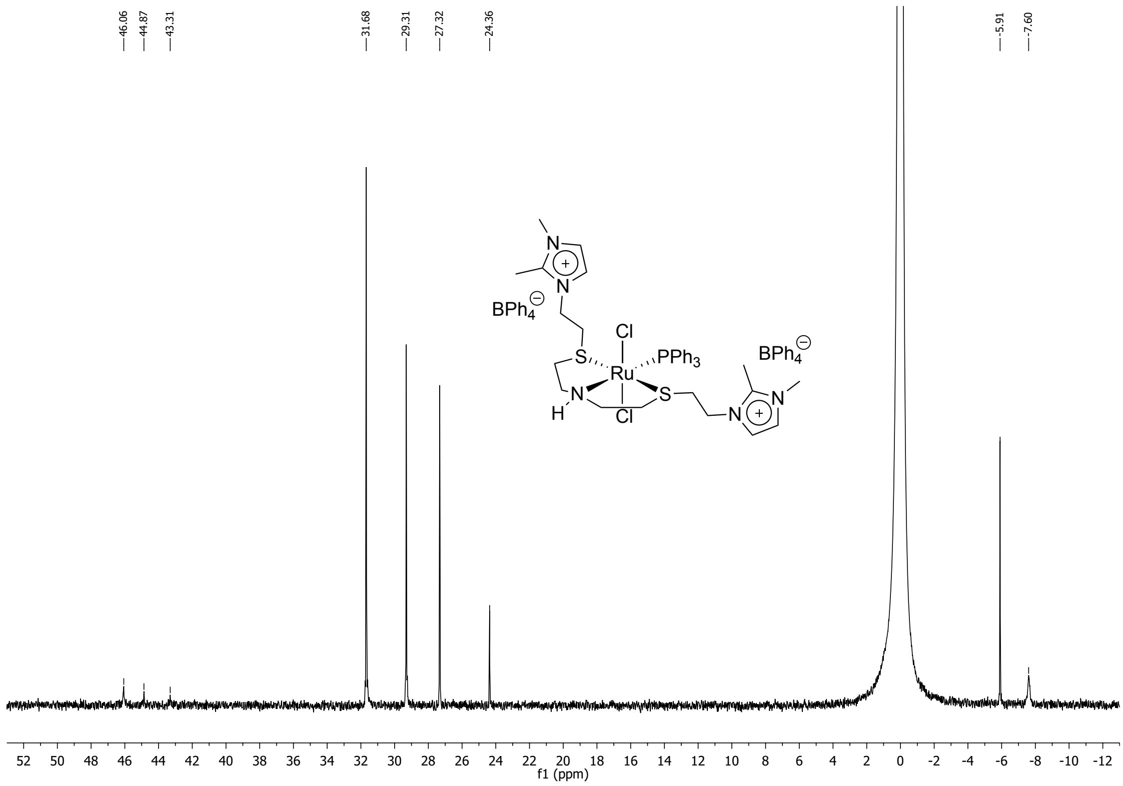


**Figure S11.** $^{31}P$ NMR analysis of the ionophilic $[RuCl_2(SNS)(PPh_3)]$ complex **2** in DMSO-$d_6$.

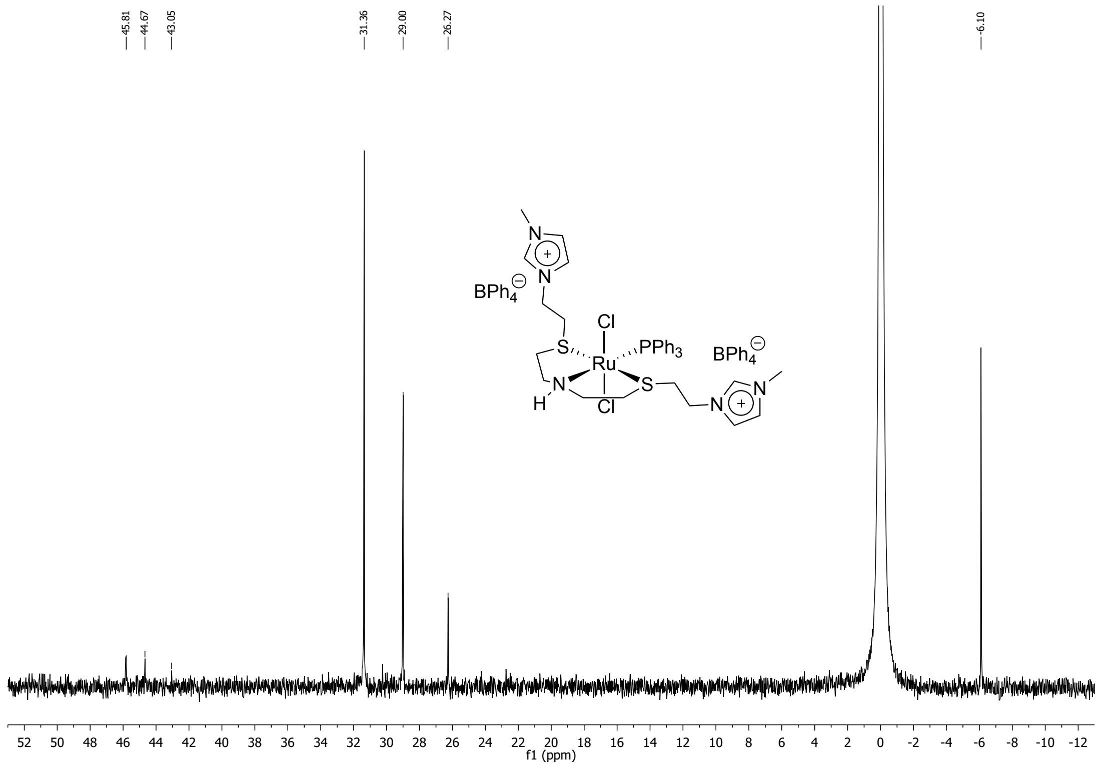


**Figure S12.** $^{31}$P NMR analysis of the ionophilic [$RuCl_2$(SNS)($PPh_3$)] complex **3** in DMSO-$d_6$.

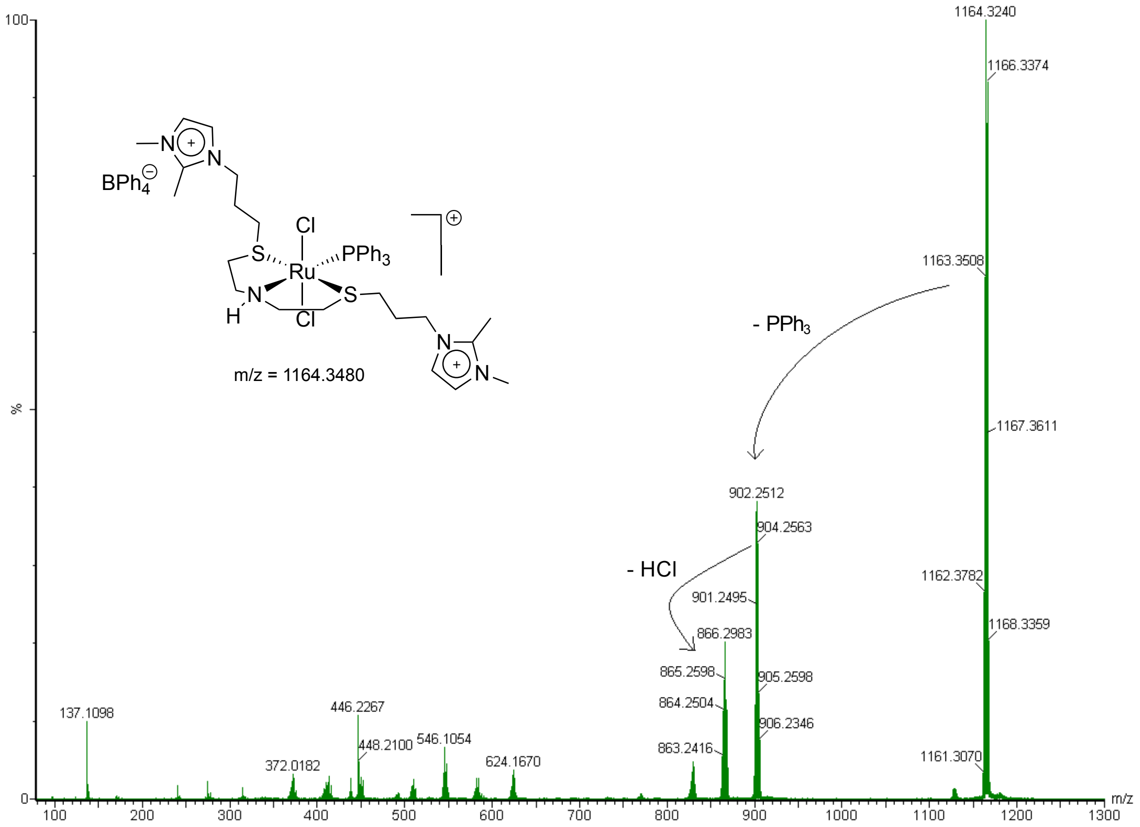


**Figure S13.** ESI(+)-MS/MS analysis of the signal at m/z = 1164 for the ionophilic [$RuCl_2$(SNS)($PPh_3$)] complex **1**.

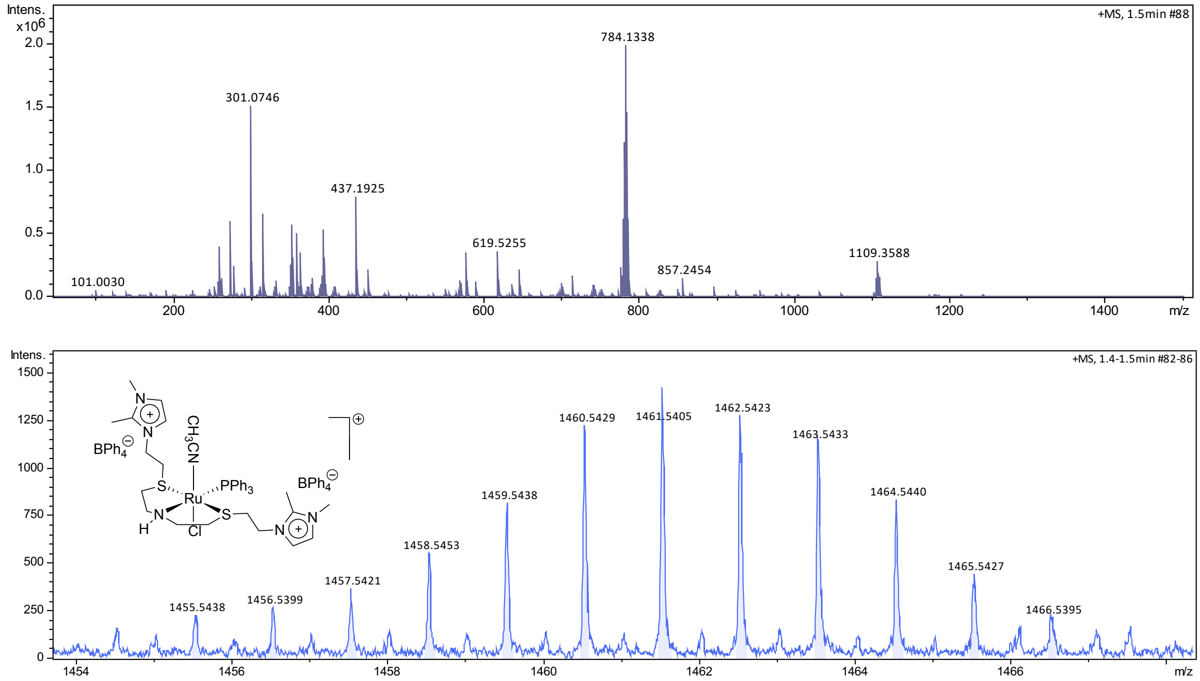


**Figure S14.** ESI(+)-MS of the ionophilic [$RuCl_2$(SNS)($PPh_3$)] complex **2**. The signal at m/z = 1461 is referred to the indicated metal species.

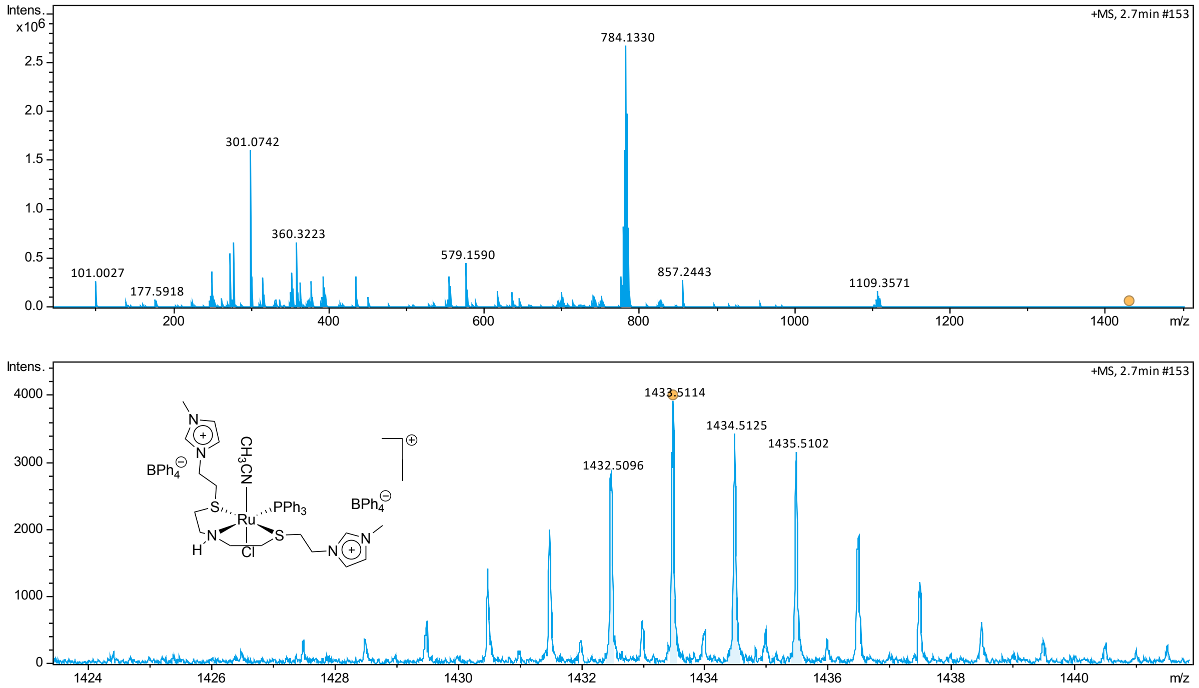


**Figure S15.** ESI(+)-MS of the ionophilic [$RuCl_2$(SNS)($PPh_3$)] complex **3**. The signal at m/z = 1433 is referred to the indicated metal species.

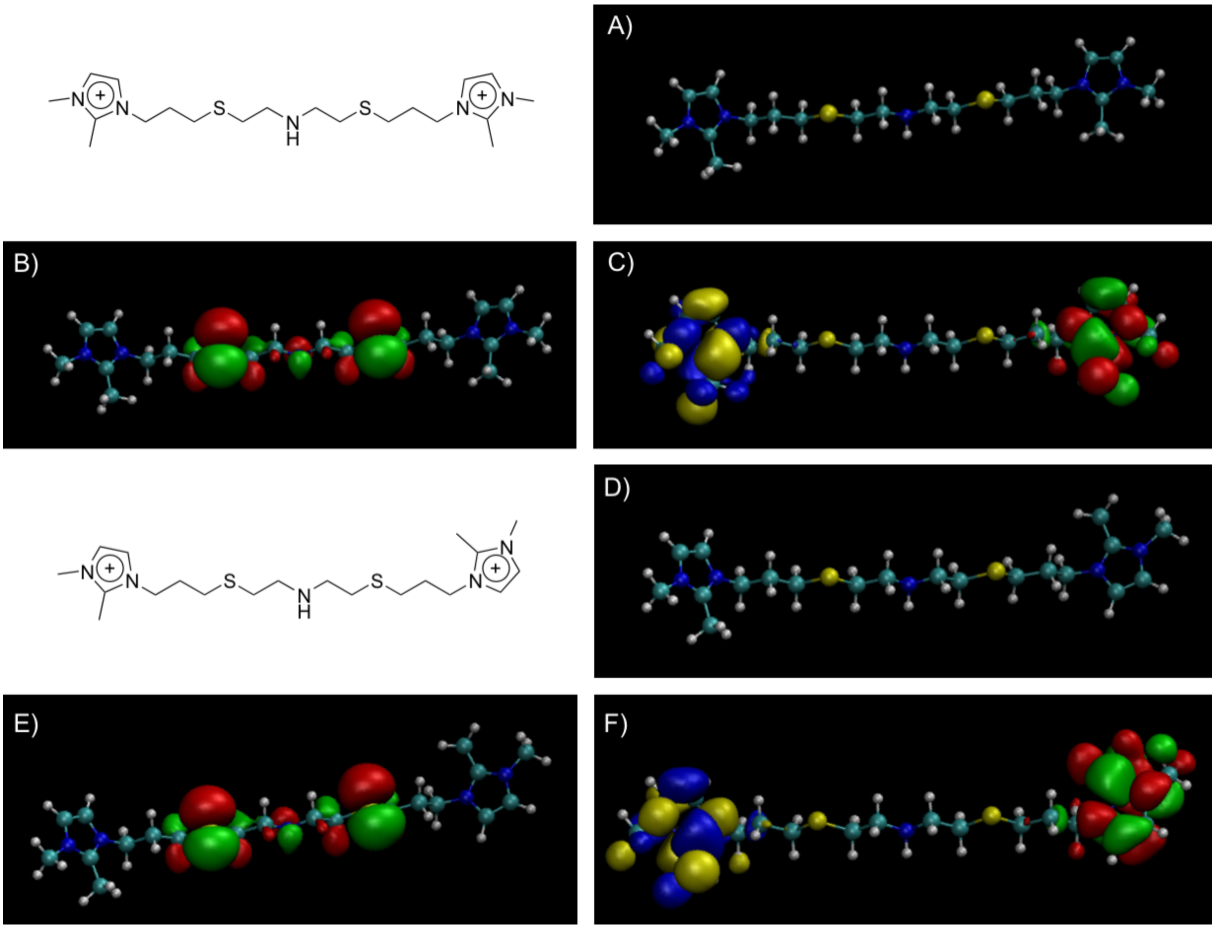


**Figure S16.** Theoretical calculations for the SNS ligand (cationic part) assuming *syn* and *anti* positions. A) and D) Optimized molecular geometry; B) and E) HOMO (isosurface 0.02); C) and F) LUMOs (isosurface 0.02): green/red LUMO and yellow/blue LUMO+1. The LUMO and LUMO+1 are energetically equivalent.

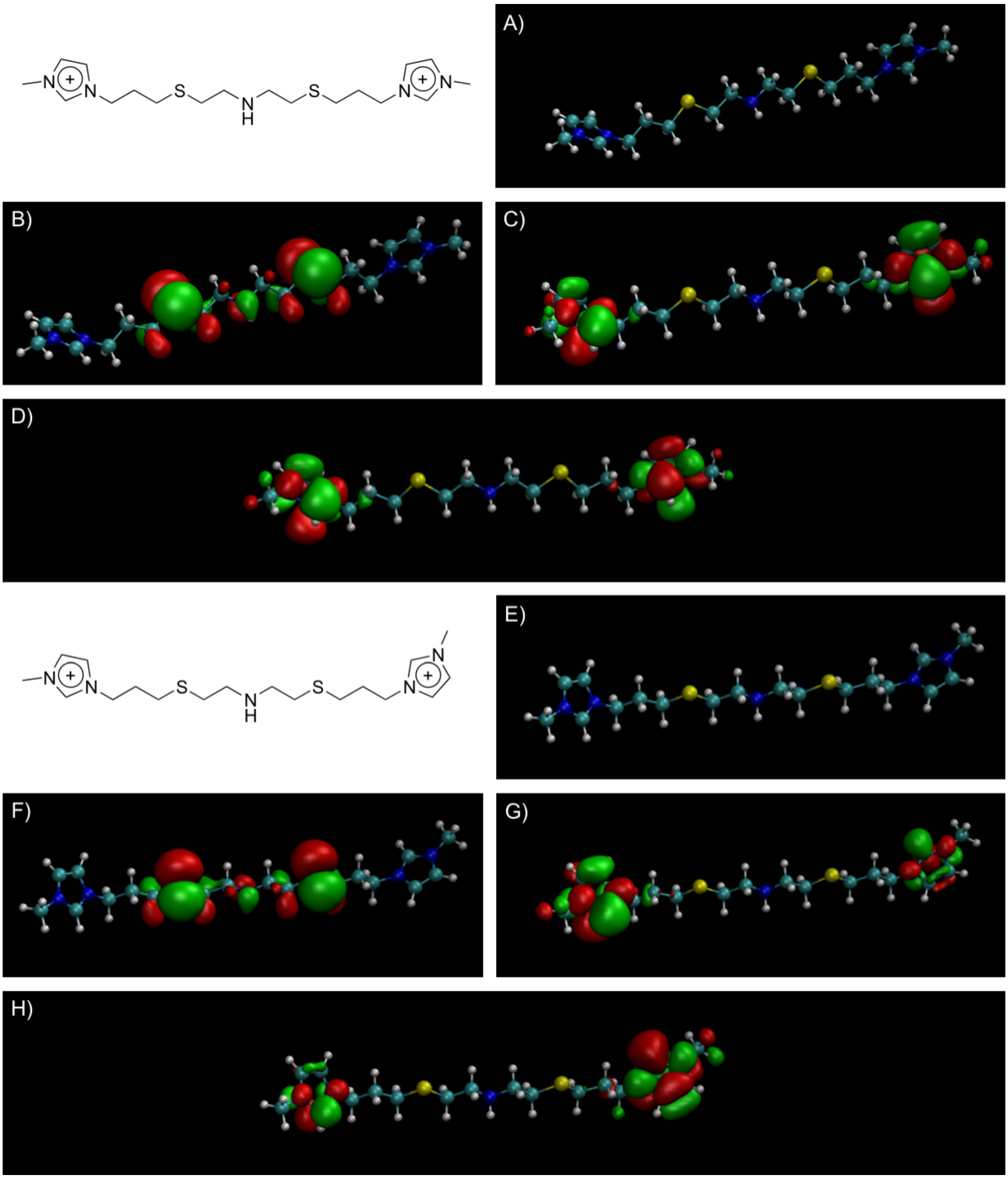


**Figure S17.** Theoretical calculations for a derived SNS ligand (cationic part) assuming *syn* and *anti* positions. A) and E) Optimized molecular geometry; B) and F) HOMO (isosurface 0.02); C) and G) LUMO (isosurface 0.02); D) and H) LUMO+1 (isosurface 0.02). The LUMO and LUMO+1 are energetically equivalent.

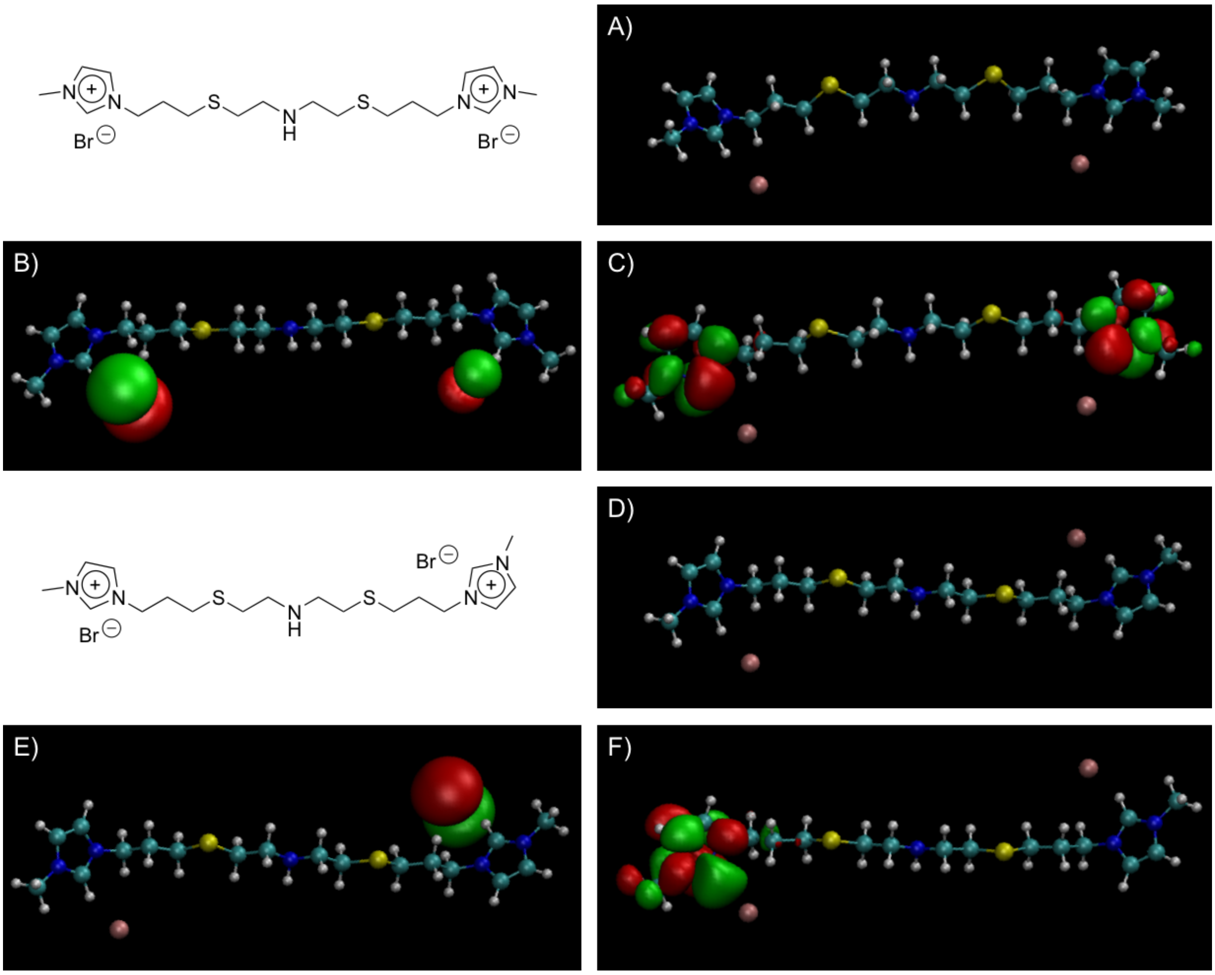


**Figure S18.** Theoretical calculations for a derived SNS ligand containing the ion pair (bromide as anion) assuming *syn* and *anti* positions. A) and D) Optimized molecular geometry; B) and E) HOMO (isosurface 0.02); C) and F) LUMO (isosurface 0.02).

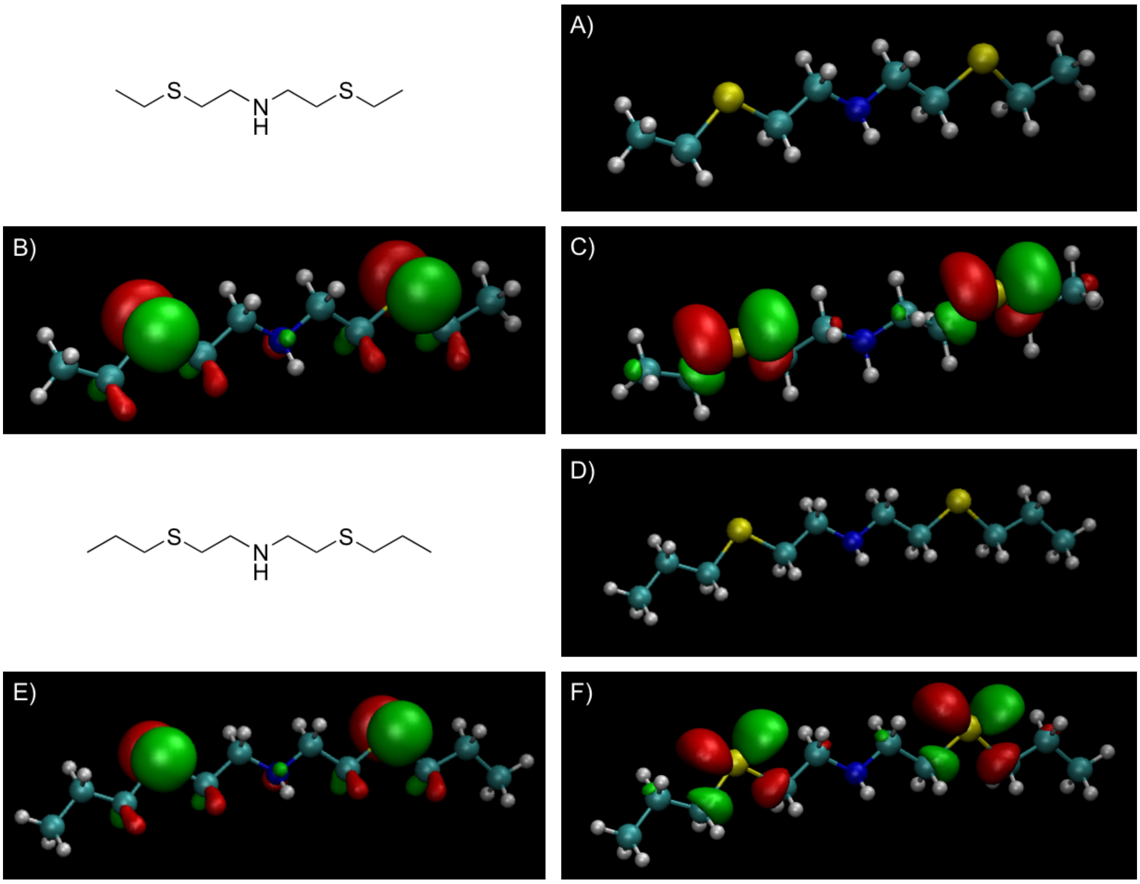


**Figure S19.** Theoretical calculations for classical non-ionophilic SNS ligands. A) and D) Optimized molecular geometry; B) and E) HOMO (isosurface 0.04); C) and F) LUMO (isosurface 0.04).

**Table S1.** Structural parameters: bond lengths and bond angles S-N-S of the SNS molecules

| Entry | Molecule | N-S (Å) | S-N-S (°) | H-Br (Å) |
|---|---|---|---|---|
| 1 | 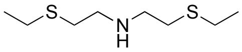 | 4.086 | 154.6 | - |
| 2 | 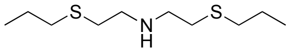 | 4.086 | 154.6 | - |
| 3 | 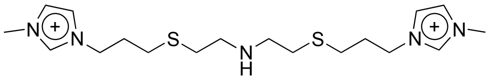 | 4.080 | 153.6 | - |
| 4 | 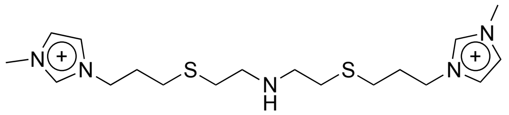 | 4.080 | 153.7 | - |
| 5 | 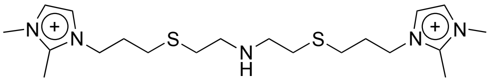 | 4.080 | 153.7 | - |
| 6 | 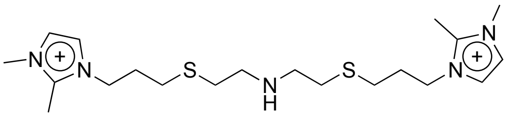 | 4.080 | 153.8 | - |
| 7 | 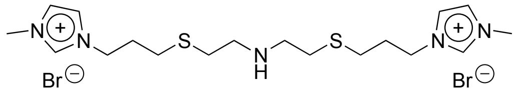 | 4.084 | 154.1 | 2.215 |
| 8 | 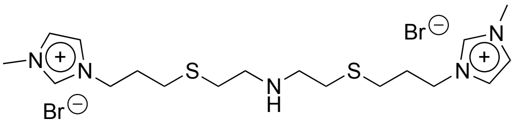 | 4.083 | 154.8 | 2.221 and 2.209 |

**Table S2.** Electrostatic properties of the SNS compounds

| Entry | Molecule | μ[a] | N | H(N)[b] | S | H2[c] | Br |
|---|---|---|---|---|---|---|---|
| 1 | 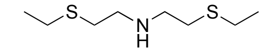 | 3.03 | -1.037 | 0.353 | -0.413 | - | - |
| 2 | 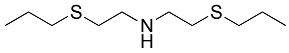 | 2.96 | -1.068 | 0.355 | -0.436 | - | - |
| 3 | 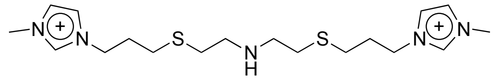 | 3.83 | -1.050 | 0.367 | -0.399 | 0.232 | - |
| 4 | 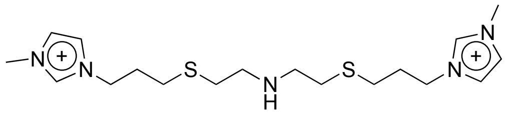 | 3.71 | -1.053 | 0.362 | -0.400 | 0.234 | - |
| 5 | 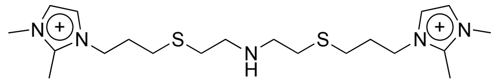 | 3.01 | -1.057 | 0.366 | -0.404 | 0.126[d] | - |
| 6 | 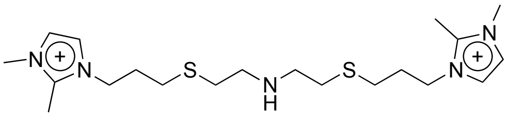 | 2.96 | -1.061 | 0.367 | -0.397 | 0.127[d] and 0.136[d] | - |
| 7 | 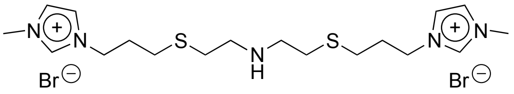 | 19.94 | -1.065 | 0.362 | -0.430 | 0.115 | -0.772 |
| 8 | 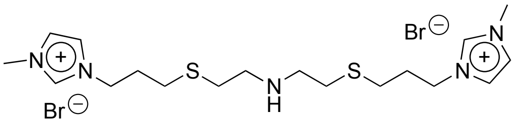 | 10.13 | -1.055 | 0.358 | -0.430 | 0.107 | -0.769 |

[a]Dipole (D): the remaining columns are point charges in units of e. [b]Hydrogen bonded to nitrogen. [c]Hydrogen bonded to the C2 of the imidazolium ring. [d]Sum of methyl charges.

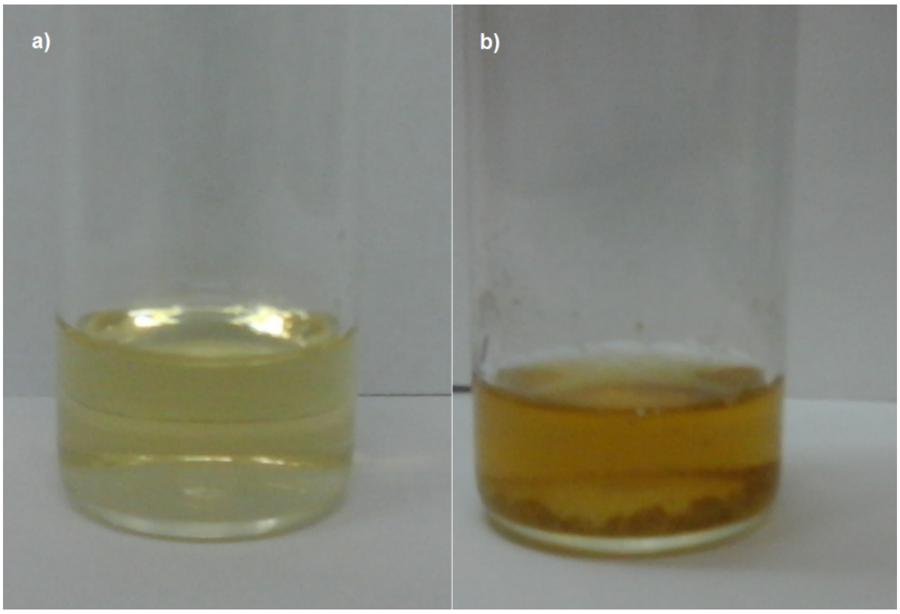


**Figure S20.** Images of the reaction mixture using acetonitrile as solvent (a) before and (b) after hydrogenation of $CO_2$.

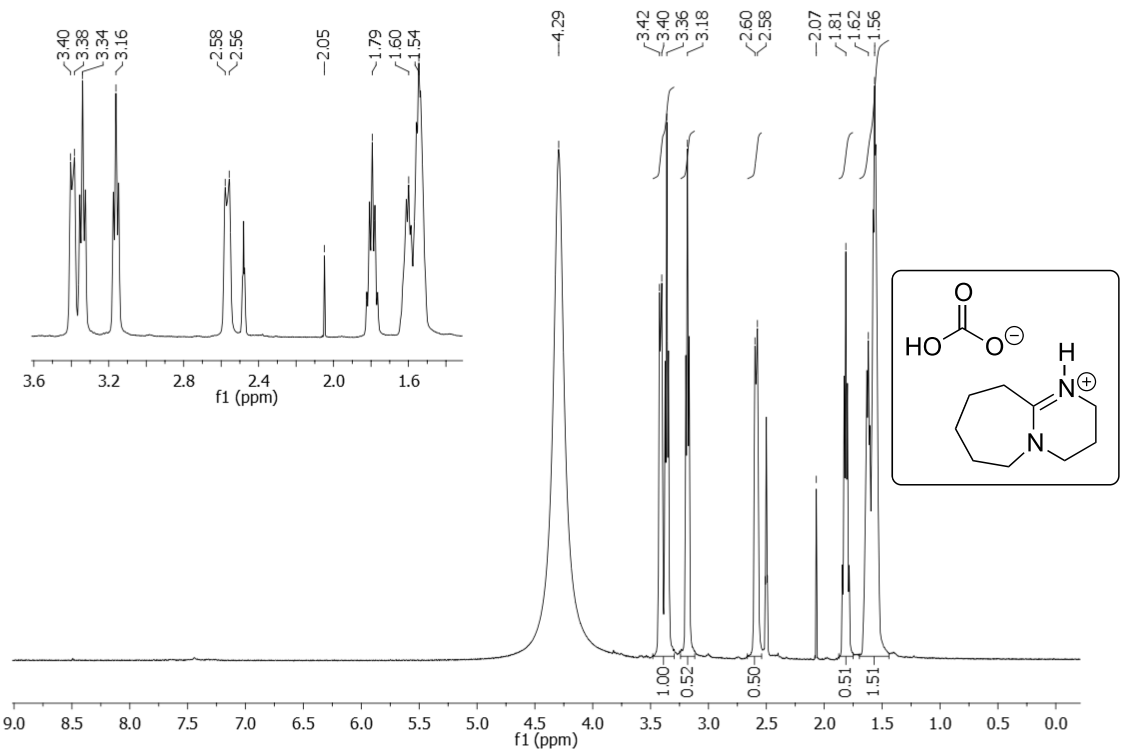


**Figure S21.** $^1$H NMR spectrum of the [DBUH][$HCO_3$] in DMSO-$d_6$.

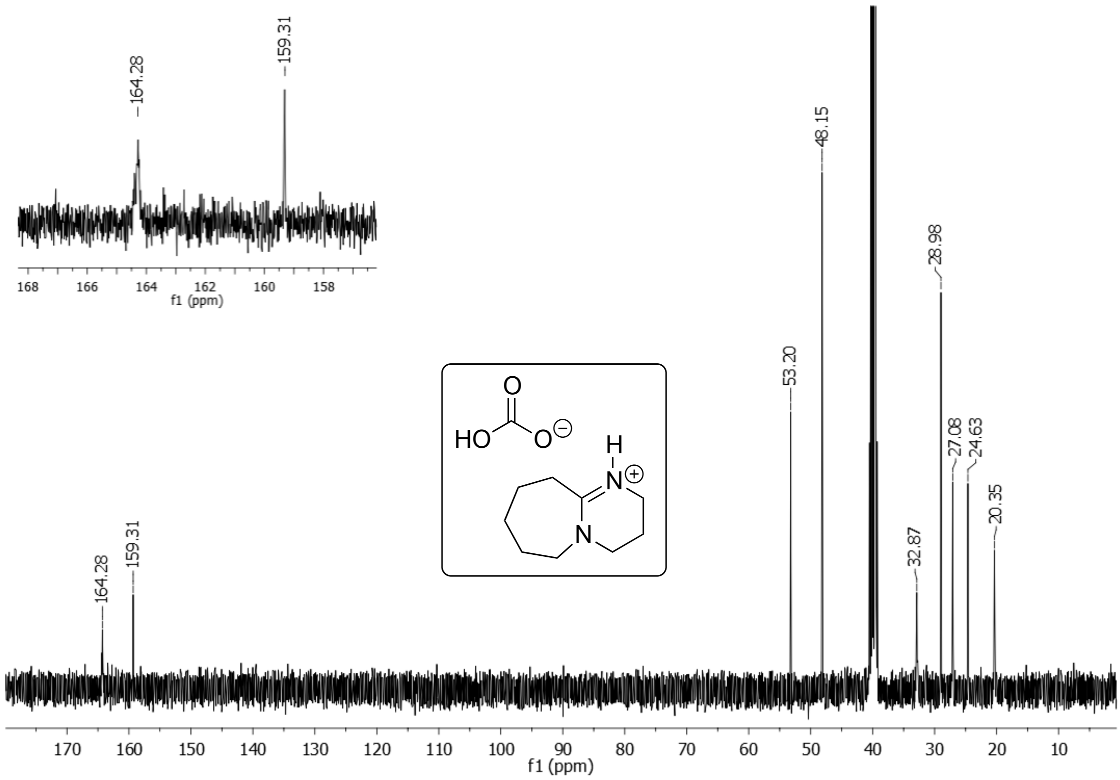


**Figure S22.** $^{13}C$ NMR spectrum of the [DBUH][$HCO_3$] in DMSO-$d_6$.

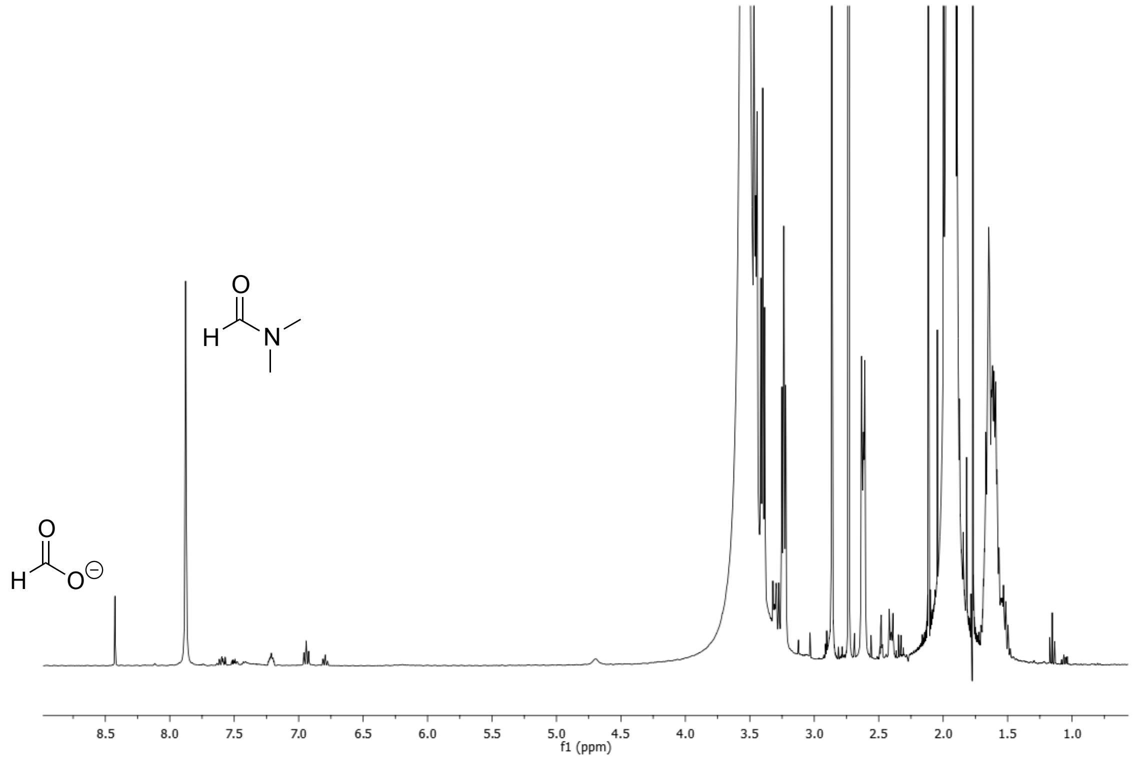


**Figure S23.** Typical $^{1}$H NMR spectrum after the hydrogenation of $CO_2$ in organic solvents. DMF was used as internal standard.

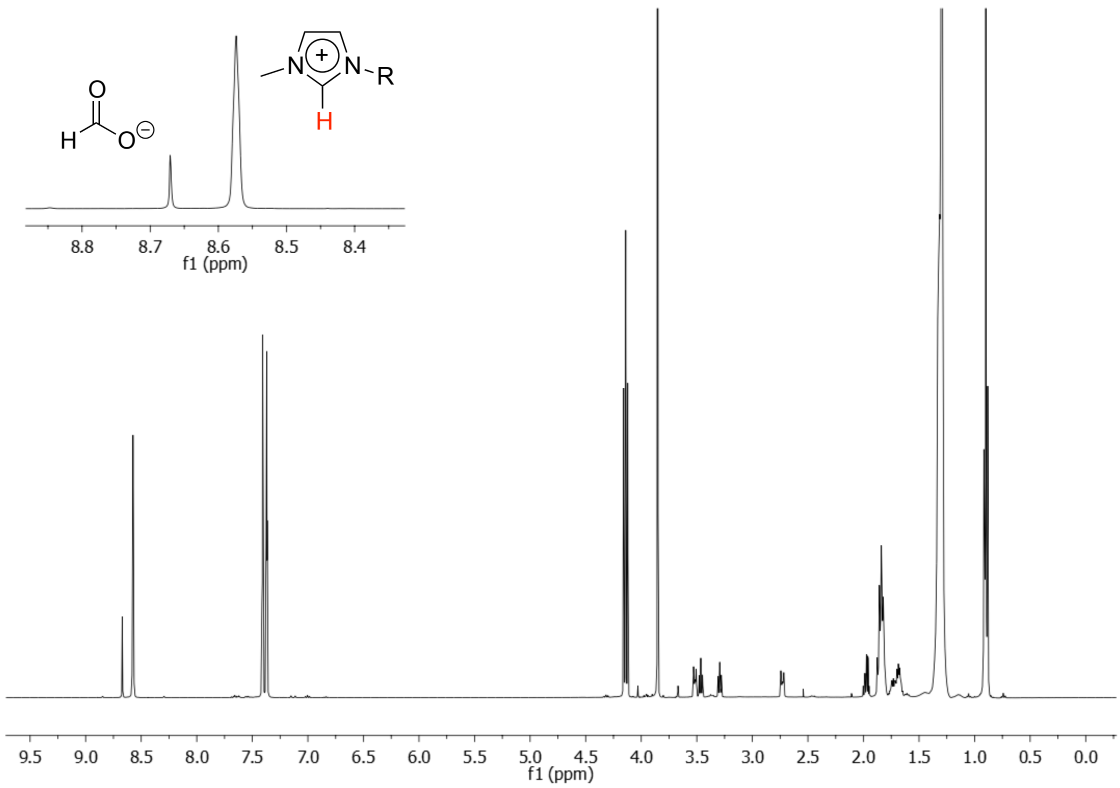


**Figure S24.** Typical $^{1}$H NMR spectrum after the hydrogenation of $CO_2$ in ionic liquids.

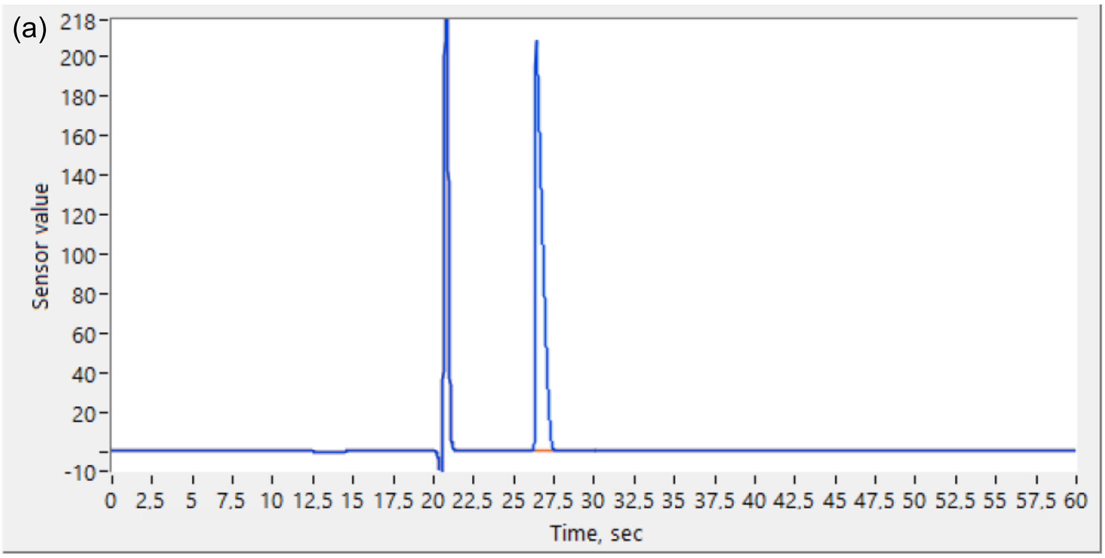


| Compound name | Quantity | Retention time | Peak area | Peak height | Peak width |
|---|---|---|---|---|---|
| Carbondioxide | 26.9329 % | 26.39 sec | 102.3208 | 207.0185 | 0.47 sec |

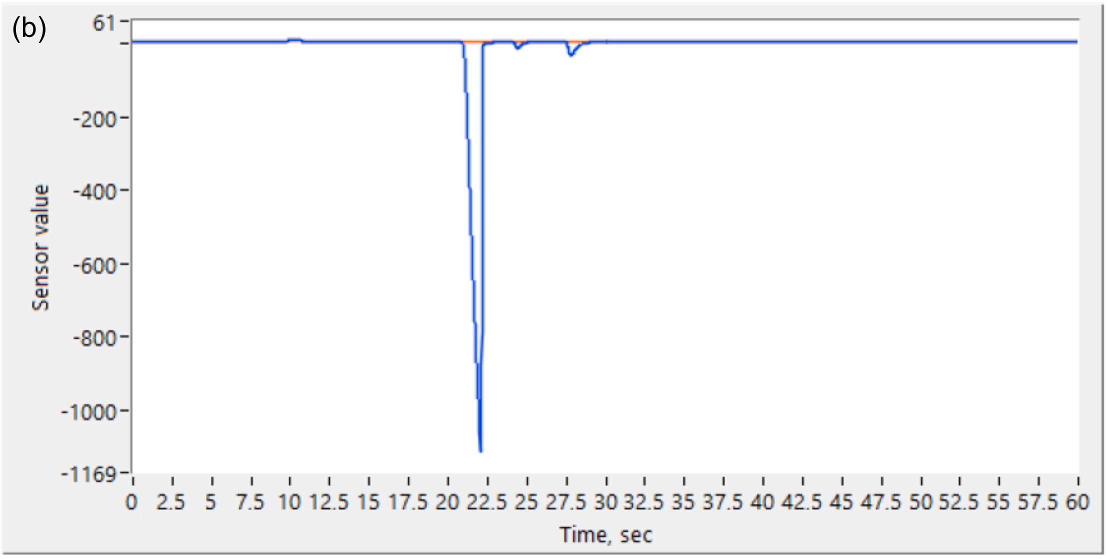


| Compound name | Quantity | Retention time | Peak area | Peak height | Peak width |
|---|---|---|---|---|---|
| Hydrogen | 46.2095 % | 22.09 sec | 704.0189 | 1116.4631 | 0.64 sec |
| Oxygen | 3.8860 % | 24.40 sec | 6.9612 | 19.1326 | 0.30 sec |
| Nitrogen | 14.5569 % | 27.78 sec | 21.1313 | 36.0679 | 0.51 sec |

**Figure S25.** GC-TCD analysis of the gas phase from $CO_2$ hydrogenation catalyzed by the ionophilic [$RuCl_2$(SNS)($PPh_3$)] complex **1** in $CH_3CN$/THF at 80 °C for 5 h. (a) Analysis showing the presence of $CO_2$ and (b) the presence of $H_2$.

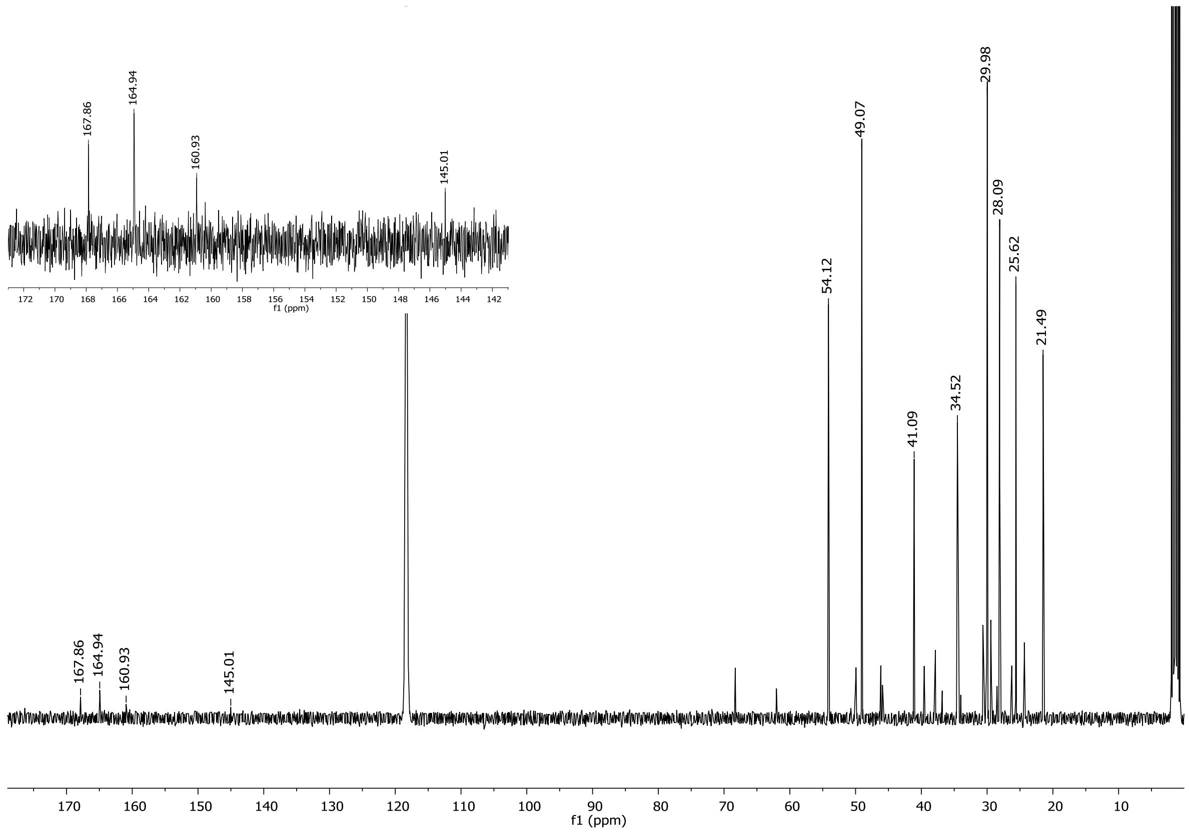


**Figure S26.** $^{13}C$ NMR spectrum in $CD_3CN$ of the solid formed in the reaction with a large excess of DBU (entry 20, Table 1).

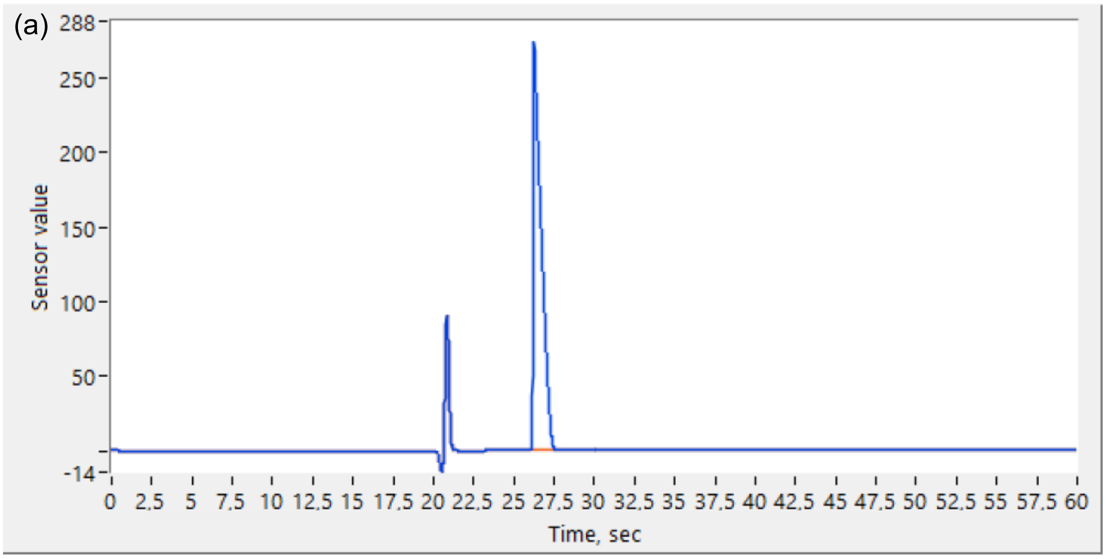


| Compound name | Quantity | Retention time | Peak area | Peak height | Peak width |
|---|---|---|---|---|---|
| Carbondioxide | 41.9043 % | 26.23 sec | 159.1985 | 273.3419 | 0.56 sec |

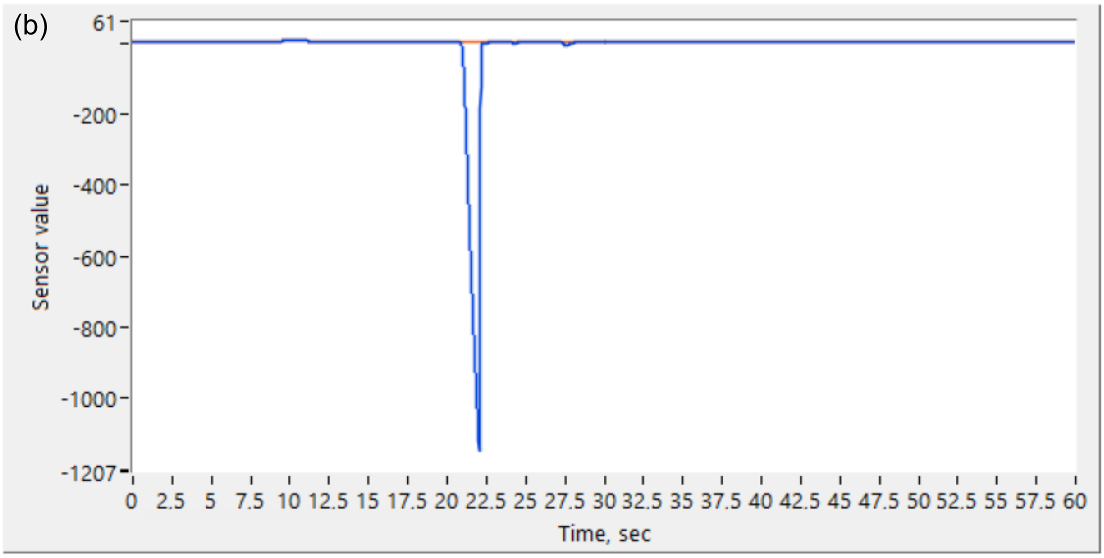


| Compound name | Quantity | Retention time | Peak area | Peak height | Peak width |
|---|---|---|---|---|---|
| Hydrogen | 47.8756 % | 22.06 sec | 729.4041 | 1151.3805 | 0.65 sec |
| Oxygen | 0.9635 % | 24.26 sec | 1.7259 | 4.2930 | 0.33 sec |
| Nitrogen | 3.3271 % | 27.59 sec | 4.8298 | 9.5040 | 0.44 sec |

**Figure S27.** GC-TCD analysis of the gas phase from $CO_2$ hydrogenation catalyzed by the ionophilic $[RuCl_2(SNS)(PPh_3)]$ complex **1** in $[BMIm][NTf_2]$ at 80 °C for 5 h. (a) Analysis showing the presence of $CO_2$ and (b) the presence of $H_2$.

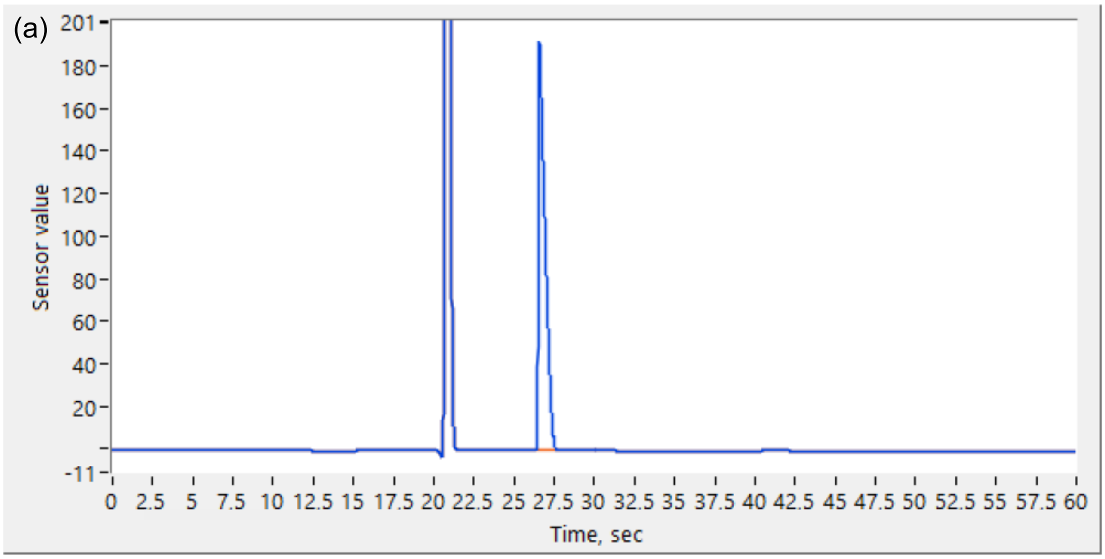


| Compound name | Quantity | Retention time | Peak area | Peak height | Peak width |
|---|---|---|---|---|---|
| Carbondioxide | 24.2527 % | 26.57 sec | 92.1384 | 191.8483 | 0.46 sec |

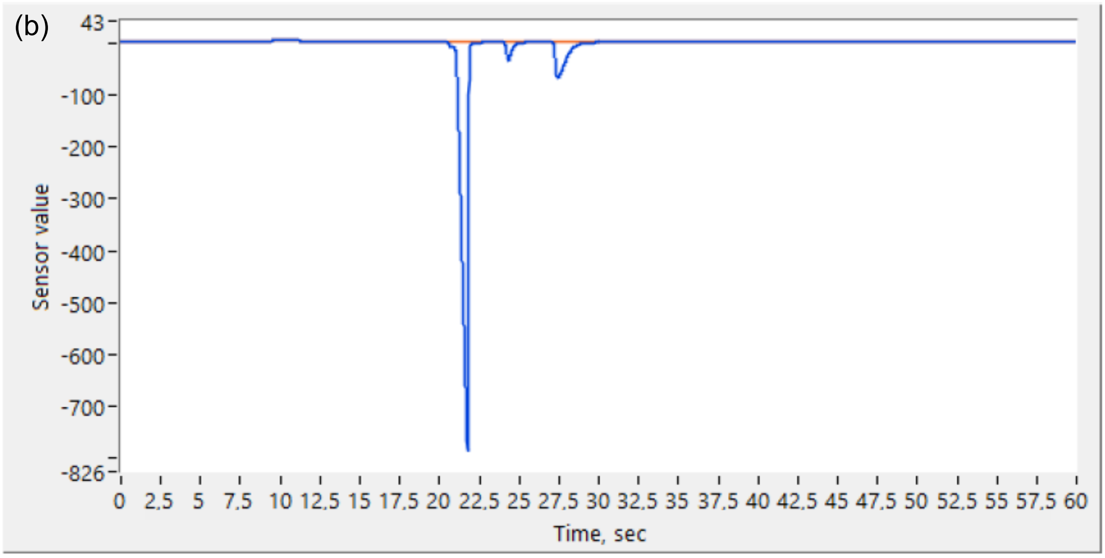


| Compound name | Quantity | Retention time | Peak area | Peak height | Peak width |
|---|---|---|---|---|---|
| Hydrogen | 22.4544 % | 21.77 sec | 342.1022 | 788.0746 | 0.43 sec |
| Oxygen | 7.6551 % | 24.33 sec | 13.7130 | 35.9194 | 0.32 sec |
| Nitrogen | 36.1723 % | 27.43 sec | 52.5088 | 68.9544 | 0.68 sec |

**Figure S28.** GC-TCD analysis of the gas phase from formic acid dehydrogenation catalyzed by the ionophilic [$RuCl_2$(SNS)($PPh_3$)] complex **1** in the presence of an amine-functionalized imidazolium IL at 80 °C for 20 h. (a) Analysis showing the presence of $CO_2$ and (b) the presence of $H_2$.

**Table S3.** Comparison of catalytic systems reported in the literature with the present study for $CO_2$ hydrogenation

| Entry | Catalyst | Pressure/ $CO_2$:$H_2$ (bar) | Temperature (°C) | Time (h) | Product | Yield (%) | Ref. |
|---|---|---|---|---|---|---|---|
| 1 | [$RuCl_2$(SNS)($PPh_3$)] **1-3** | 40-60/1:1 | 80 | 2-5 | Formate | 80-100 | This work |
| 2 | [$RuH_2$($PMe_3$)$_4$] | 205/1.4:1 | 50 | 1 | Formic acid | 45[a] | [1] |
| 3 | [RuCl(OAc)($PMe_3$)$_4$] | 40/1:1 | 50 | 10 | Formic acid | 1.68[b] | [2] |
| 4 | [RuHCl(PNP)(CO)] | 40/1:3 | 120 | 1 | Formate | 1.1[c] | [3] |
| 5 | [Ru(PPP)(TMM)] | 80/1:3 | 140 | 24 | Methanol | [d] | [4] |
| 6 | [RuHCl(PNN)(CO)] | [e] | 135 | 19 | Methanol | 92 | [5] |
| 7 | [RuCl($PhCO_2$)(PNP)($PPh_3$)] | 120/1:2 | 60 | 16 | Formic acid | [f] | [6] |
| 8 | [RuH(H-$BH_3$)(PNP)(CO)] | 75/1:3 | 95-155 | 36 | Methanol | [g] | [7] |
| 9 | [RuHCl(PNP)(CO)] | 75/1:3 | 95-155 | 36 | Methanol | [h] | [7] |
| 10 | [$RuCl_2$(SNS)(CO)] | 75/1:3 | 95-155 | 36 | --- | --- | [7] |
| 11 | [RuBr(CNC)$(CO)_2$][Br] | 60/1:1 | 100-140 | 72 | Formic acid | [i] | [8] |
| 12 | [RuH(PNP)(CO)($PPh_3$)][Cl] | 40/1:1 | 90 | 16 | Formate | 68 | [9] |
| 13 | [$RuH_2$(PNP)(CO)] | 30/1:2 | 25 | 18 | Formic acid | >95 | [10] |
| 14 | [$RuCl_2$(PNP)(NO)][X] | 30/1:2 | 30-40 | 18 | Formic acid | 56-75[j] | [11] |

[a]Estimated based on the reported TOF value and the amine-to-catalyst molar ratio used. [b]Determined as the molar ratio of formic acid to amine produced in the reaction with $C_6F_5OH$ as an additive. [c]Determined as acid-to-amine ratio produced in the reaction. [d]Determined as 2.8 mmol of methanol produced in the reaction using one equivalent of $HNTf_2$. [e]$CO_2$ capture (1-3 bar) was first carried out in the presence of an aminoalcohol to form oxazolidinone, which was subsequently hydrogenated (60 bar $H_2$) to methanol. [f]Determined as a formic acid concentration of 0.33 mol $L^{-1}$ (which increased to 1.27 mol $L^{-1}$ in the presence of acetate buffer). [g]Determined as 9 mmol of methanol produced in the reaction. A methanol yield of 79% was achieved using $CO_2$ from air under 50 bar $H_2$ at 155 °C for 55 h. [h]Determined as 9.1 mmol of methanol produced in the reaction catalyzed by the Ru complex with R = H, since the catalyst with R = Me was not active for methanol formation. [i]Determined as a formic acid concentration of up to 0.49 mol $L^{-1}$. [j]Formic acid yield >95% was achieved using 0.5 mol% [Ru][$BF_4$] at 15 bar ($CO_2$: $H_2$ 1:2) and 30 °C for 18 h.

## References

(1) Jessop, P. G.; Ikariya, T.; Noyori, R. Homogeneous catalytic hydrogenation of supercritical carbon dioxide. *Nature* **1994**, *368* (6468), 231-233.

(2) Munshi, P.; Main, A. D.; Linehan, J. C.; Tai, C. C.; Jessop, P. G. Hydrogenation of carbon dioxide catalyzed by ruthenium trimethylphosphine complexes: The accelerating effect of certain alcohols and amines. *J. Am. Chem. Soc.* **2002**, *124* (27), 7963-7971.

(3) Filonenko, G. A.; van Putten, R.; Schulpen, E. N.; Hensen, E. J. M.; Pidko, E. A. Highly Efficient Reversible Hydrogenation of Carbon Dioxide to Formates Using a Ruthenium PNP-Pincer Catalyst. *ChemCatChem* **2014**, *6* (6), 1526-1530.

(4) Wesselbaum, S.; Moha, V.; Meuresch, M.; Brosinski, S.; Thenert, K. M.; Kothe, J.; vom Stein, T.; Englert, U.; Hölscher, M.; Klankermayer, J.; Leitner, W. Hydrogenation of carbon dioxide to methanol using a homogeneous ruthenium-Triphos catalyst: from mechanistic investigations to multiphase catalysis. *Chem. Sci.* **2015**, *6* (1), 693-704.

(5) Khusnutdinova, J. R.; Garg, J. A.; Milstein, D. Combining Low-Pressure $CO_2$ Capture and Hydrogenation To Form Methanol. *ACS Catal.* **2015**, *5* (4), 2416-2422.

(6) Rohmann, K.; Kothe, J.; Haenel, M. W.; Englert, U.; Hölscher, M.; Leitner, W. Hydrogenation of $CO_2$ to Formic Acid with a Highly Active Ruthenium Acriphos Complex in DMSO and DMSO/Water. *Angew. Chem. Int. Ed.* **2016**, *55* (31), 8966-8969.

(7) Kothandaraman, J.; Goeppert, A.; Czaun, M.; Olah, G. A.; Prakash, G. K. S. Conversion of $CO_2$ from Air into Methanol Using a Polyamine and a Homogeneous Ruthenium Catalyst. *J. Am. Chem. Soc.* **2016**, *138* (3), 778-781.

(8) Weilhard, A.; Argent, S. P.; Sans, V. Efficient carbon dioxide hydrogenation to formic acid with buffering ionic liquids. *Nat. Commun.* **2021**, *12* (1), 231.

(9) Tossaint, A. S.; Rebreyend, C.; Sinha, V.; Weber, M.; Canossa, S.; Pidko, E. A.; Filonenko, G. A. Two step activation of Ru-$PN^3P$ pincer catalysts for $CO_2$ hydrogenation. *Catal. Sci. Technol.* **2022**, *12* (9), 2972-2977.

(10) Piccirilli, L.; Rabell, B.; Padilla, R.; Riisager, A.; Das, S.; Nielsen, M. Versatile $CO_2$ Hydrogenation–Dehydrogenation Catalysis with a Ru–PNP/Ionic Liquid System. *J. Am. Chem. Soc.* **2023**, *145* (10), 5655-5663.

(11) Correia, J. T. M.; Nori, V.; Jørgensen, M. S. B.; Nikol, A. T.; Nielsen, M. Ru-Nitrosyl Complex Salts as Efficient Catalysts for the Reversible $CO_2$ Hydrogenation/FA Dehydrogenation in Ionic Liquids. *JACS Au* **2025**, *5* (5), 2114-2122.
